# Chemotherapy-induced oral mucositis is associated with detrimental bacterial dysbiosis

**DOI:** 10.1186/s40168-019-0679-5

**Published:** 2019-04-25

**Authors:** Bo-Young Hong, Takanori Sobue, Linda Choquette, Amanda K. Dupuy, Angela Thompson, Joseph A. Burleson, Andrew L. Salner, Peter K. Schauer, Pujan Joshi, Evan Fox, Dong-Guk Shin, George M. Weinstock, Linda D. Strausbaugh, Anna Dongari-Bagtzoglou, Douglas E. Peterson, Patricia I. Diaz

**Affiliations:** 1 Department of Oral Health and Diagnostic Sciences, School of Dental Medicine, UConn Health, 263 Farmington Ave, Farmington, CT 06030-1710 USA; 2 Jackson Laboratory for Genomic Medicine, Farmington, CT USA; 3 Department of Molecular and Cell Biology, University of Connecticut, Storrs, CT USA; 4 Department of Community Medicine and Health Care, UConn Health, Farmington, CT USA; 5 Hartford Healthcare, Hartford, CT USA; 6 Department of Computer Science, University of Connecticut, Storrs, CT USA

**Keywords:** Microbiome, Cancer, Chemotherapy, Oral mucositis, Mucosal-microbial crosstalk

## Abstract

**Background:**

Gastrointestinal mucosal injury (mucositis), commonly affecting the oral cavity, is a clinically significant yet incompletely understood complication of cancer chemotherapy. Although antineoplastic cytotoxicity constitutes the primary injury trigger, the interaction of oral microbial commensals with mucosal tissues could modify the response. It is not clear, however, whether chemotherapy and its associated treatments affect oral microbial communities disrupting the homeostatic balance between resident microorganisms and the adjacent mucosa and if such alterations are associated with mucositis. To gain knowledge on the pathophysiology of oral mucositis, 49 subjects receiving 5-fluorouracil (5-FU) or doxorubicin-based chemotherapy were evaluated longitudinally during one cycle, assessing clinical outcomes, bacterial and fungal oral microbiome changes, and epithelial transcriptome responses. As a control for microbiome stability, 30 non-cancer subjects were longitudinally assessed. Through complementary in vitro assays, we also evaluated the antibacterial potential of 5-FU on oral microorganisms and the interaction of commensals with oral epithelial tissues.

**Results:**

Oral mucositis severity was associated with 5-FU, increased salivary flow, and higher oral granulocyte counts. The oral bacteriome was disrupted during chemotherapy and while antibiotic and acid inhibitor intake contributed to these changes, bacteriome disruptions were also correlated with antineoplastics and independently and strongly associated with oral mucositis severity. Mucositis-associated bacteriome shifts included depletion of common health-associated commensals from the genera *Streptococcus*, *Actinomyces*, *Gemella*, *Granulicatella*, and *Veillonella* and enrichment of Gram-negative bacteria such as *Fusobacterium nucleatum* and *Prevotella oris*. Shifts could not be explained by a direct antibacterial effect of 5-FU, but rather resembled the inflammation-associated dysbiotic shifts seen in other oral conditions. Epithelial transcriptional responses during chemotherapy included upregulation of genes involved in innate immunity and apoptosis. Using a multilayer epithelial construct, we show mucositis-associated dysbiotic shifts may contribute to aggravate mucosal damage since the mucositis-depleted *Streptococcus salivarius* was tolerated as a commensal, while the mucositis-enriched *F. nucleatum* displayed pro-inflammatory and pro-apoptotic capacity.

**Conclusions:**

Altogether, our work reveals that chemotherapy-induced oral mucositis is associated with bacterial dysbiosis and demonstrates the potential for dysbiotic shifts to aggravate antineoplastic-induced epithelial injury. These findings suggest that control of oral bacterial dysbiosis could represent a novel preventive approach to ameliorate oral mucositis.

**Electronic supplementary material:**

The online version of this article (10.1186/s40168-019-0679-5) contains supplementary material, which is available to authorized users.

## Background

One of the most frequent complications of chemotherapy is oral mucositis, reported to affect about 75% of patients receiving high-dose conditioning chemotherapy prior to hematopoietic cell transplantation and 20 to 60% of individuals treated for solid tumors [1, 2]. Lesions present as erythema and ulceration of the non-keratinized mucosa. Although the condition is self-limiting, it can impact the delivery of optimal cancer treatment, since it is associated with clinically significant pain, compromised nutrition, prolonged hospitalization, bloodstream infections and antineoplastic dose reductions [3, 4]. Different approaches to prevent or treat oral mucositis have been largely ineffective as there is insufficient knowledge on its pathophysiology [5].

Oral mucositis primarily results from the cytotoxic effects of chemotherapeutics on the rapidly dividing oral epithelium, with other areas along the gastrointestinal tract also commonly affected. Antineoplastics exert their cytotoxic effects by a variety of mechanisms leading to impaired DNA replication and repair, cell-cycle arrest, DNA damage, and cell death [6, 7]. However, the specific downstream cellular events mediating oral epithelial damage remain poorly characterized. It has been proposed that oral microbiome communities, which live in constant cross-talk with the adjacent mucosal tissues, could contribute to the development of mucositis [8, 9]. Evidence from animal models of intestinal mucositis suggests it is plausible that epithelial-microbiome cross-talk along the gastrointestinal track modulates susceptibility to mucositis, with resident microbial commensals shown to be necessary for irinotecan-dependent intestinal mucosal injury [10]. Also, mice deficient in the innate immunity activator Toll-like receptor 2 show increased susceptibility to methotrexate-induced intestinal mucositis, an indication that microbial signaling could modify the severity of the response [11].

It is not completely clear how chemotherapy alters the oral environment. Various studies in humans using microbiological techniques with limited power of detection indicate the oral microbiota changes during cancer treatment [12, 13]. However, there is a paucity of longitudinal, well-controlled studies that use highly sensitive high throughput sequencing to characterize the oral microbiome during chemotherapy. Disruption of microbiome communities, allowing growth of pathobionts, could negatively impact the ability of mucosal tissues to remain intact during an antineoplastic challenge. The plausibility of this hypothesis was demonstrated in an animal model of intestinal mucositis, in which the antineoplastic cisplatin induced significant changes in the intestinal microbiome, but restoration of the gut microbiota through fecal-pellet gavage promoted healing of cisplatin-induced mucositis [14]. It is possible that similar events take place in the oral cavity, where chemotherapy could induce microbiome disruptions that affect the susceptibility of oral tissues to mucositis.

Alterations in the oral microbiome during chemotherapy could result from impairment of the different constituents involved in maintaining these communities in a commensal state. It has been suggested that chemotherapy compromises the flow of saliva, one of the most important defense mechanisms in the oral cavity [15, 16]. Chemotherapy-associated myelosuppression could also compromise the availability of oral neutrophils, resulting in a more permissive environment for detrimental oral microorganisms. Other chemotherapy-associated treatments such as antibiotics could also affect the oral microbiome. In addition, antineoplastics have been shown to have antimicrobial activity and could potentially contribute to microbiome changes during chemotherapy [17, 18].

Here, we present results of a comprehensive longitudinal evaluation of subjects undergoing 5-fluorouracil (5-FU) or doxorubicin-based chemotherapy, characterizing changes in the bacterial and fungal oral microbiome, salivary flow rate (SFR), and oral granulocyte availability during one treatment cycle and in relation to the development of oral mucositis. As a control for microbiome stability, non-cancer subjects were also longitudinally sampled. We found that oral mucositis correlated with the dose of 5-FU, docetaxel, and cisplatin, increased salivary flow, and greater oral granulocyte presence. Chemotherapy disrupted the oral bacteriome with these shifts showing a strong correlation with oral mucositis severity. Shifts were not specific to mucositis lesions but rather affected the oral cavity as a whole. Microbiome shifts associated with mucositis were independent from the effect of antibiotics and acid inhibitors. Although shifts correlated with antineoplastic doses, and 5-FU showed potential to modify directly the microbiome, mucositis-associated shifts were unlikely the result of a selective antibacterial action of 5-FU, as commensals depleted during mucositis were resistant to physiologically relevant drug concentrations and *Fusobacterium nucleatum*, a species enriched during mucositis, was sensitive to 5-FU. Bacteria with a longitudinal change in abundance that negatively correlated with mucositis severity included species of *Streptococcus*, *Actinomyces*, *Gemella*, *Granulicatella*, and *Veillonella*, all common in oral health, while more severe mucositis correlated with enrichment of Gram-negatives previously associated with other oral inflammatory conditions [19]. These results are consistent with mucositis-associated dysbiosis as a result of alterations in nutritional resources due to inflammation, echoing other oral conditions in which inflammatory products promote pathobiont growth, which in turn alters host responses [20]. To better understand mucosal responses during chemotherapy, we longitudinally characterized oral epithelial transcriptome changes in a subset of subjects, finding genes involved in the innate immune response and apoptosis, among others, to be upregulated. The potential for a dysbiotic microbiome to modify mucosal responses was evaluated in a 3D oral mucosa multilayered construct. In this model, we show that *Streptococcus salivarius*, a potential symbiont depleted during mucositis, is tolerated by the oral mucosa. In contrast, *Fusobacterium nucleatum* subsp. *vincentii*, associated with more severe mucositis, has pro-inflammatory and pro-apoptotic effects on oral epithelial cells. To our knowledge, this study provides the first comprehensive characterization of oral mucositis pathophysiology in humans demonstrating bacterial dysbiosis is associated with lesion severity, with such bacteriome changes showing clear potential to contribute to mucositis by promoting further epithelial damage.

## Results

### Oral mucositis is associated with 5-FU exposure

**Fig. 1:**
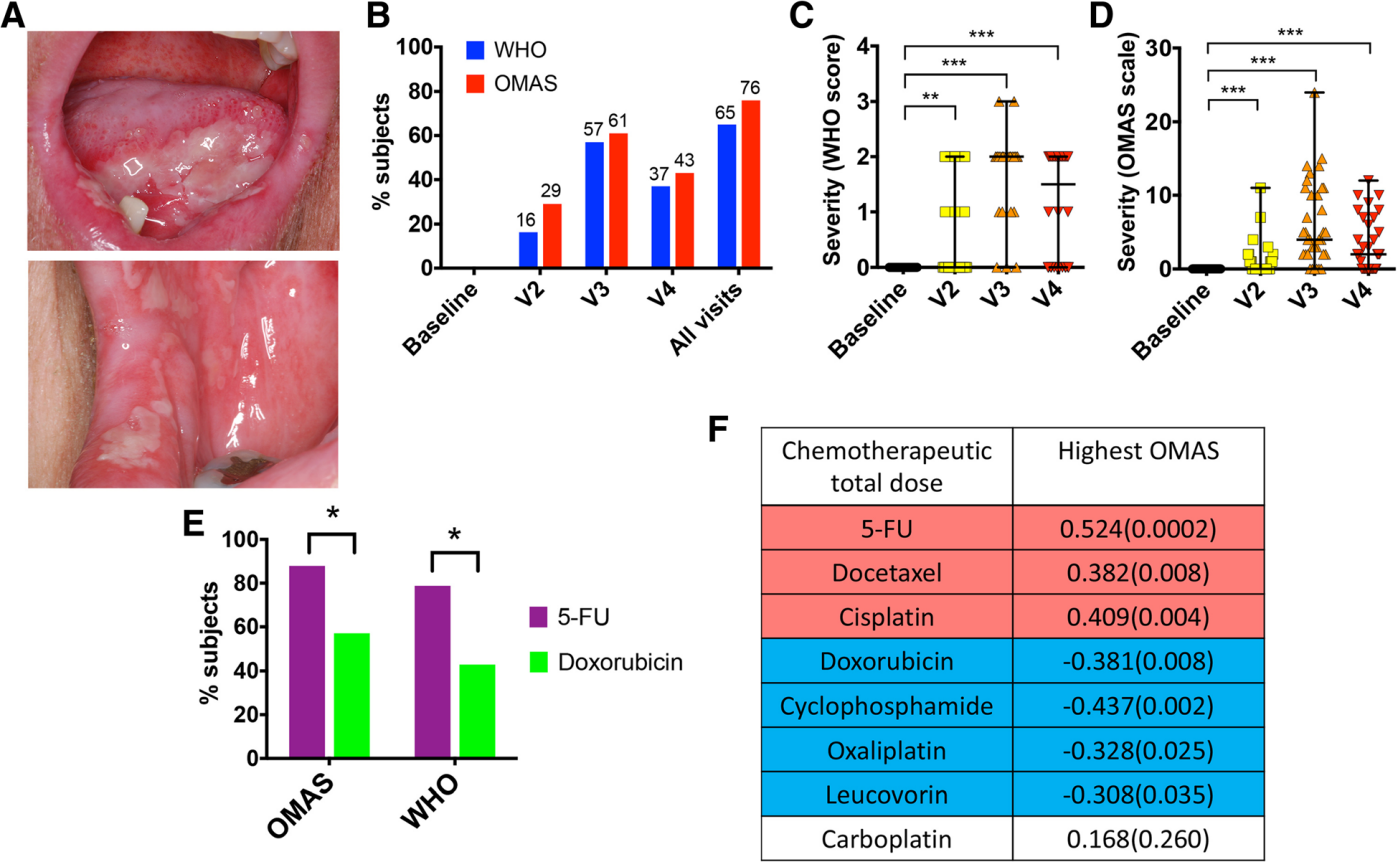
Incidence and clinical presentation of oral mucositis during chemotherapy and correlation with antineoplastics. **a** Intraoral images of a patient affected by oral mucositis. **b** Mucositis incidence according to the WHO scale, which evaluates in a categorical scale of 0 to 4 objective signs and patient-reported symptoms, and to the OMAS scale, which is based solely on objective signs of erythema and ulceration. OMAS scores reported here could range from 0 to 45 and represent the aggregated scores from nine intra-oral sites evaluated. **c**, **d** The clinical progression of mucositis in mucositis-positive subjects (*n* = 32 for the WHO scale and *n* = 37 for OMAS). Graphs show individual data points with median and range. ** indicates a *p* value < 0.01 and *** indicates a *p* value < 0.001 when comparing each time point to baseline via Wilcoxon matched-pairs signed rank tests. **e** Incidence of mucositis in subjects taking 5-FU and those on doxorubicin. * indicates a *p* value of < 0.05 when comparing incidence via chi-square. **f** Correlations between chemotherapeutic total drug doses and mucositis severity. Data represent Spearman correlation coefficients with *p* values in parenthesis. Colored cells show correlations significant after adjustment for multiple comparisons via the FDR method. Only drugs given to at least 15% of subjects were included in the analysis

To better understand the pathophysiology of oral mucositis we followed 49 subjects during one cycle of 5-FU- or doxorubicin-based chemotherapy (Additional file 1: Figure S1 and Additional file 2: Tables S1 and S2) and recorded the incidence and severity of oral mucositis. Fig. 1a shows a typical oral mucositis clinical presentation with erythema and ulceration affecting mostly the non-keratinized mucosa. As seen in Fig. 1b, mucositis occurred in 65 to 76% of subjects as measured via the WHO and OMAS scales, respectively, with mucositis severity peaking at V3 (Fig. 1c, d). Mucositis incidence was higher in subjects on the 5-FU-based regimen in comparison to those taking doxorubicin (Fig. 1e, WHO *p* = 0.016 and OMAS *p* = 0.019), and a moderate but highly significant positive correlation was seen between the dose of 5-FU and mucositis severity (Fig. 1f). The doses of docetaxel and cisplatin, which were commonly given in combination with 5-FU (Additional file 2: Table S2), also positively correlated with more severe mucositis; while other drugs given with 5-FU such as carboplatin, oxaliplatin and the chemoprotectant leucovorin showed no correlation or a negative correlation with mucositis severity (Fig. 1f).

### Oral mucositis is associated with increased salivary flow and greater oral neutrophil presence

**Fig. 2:**
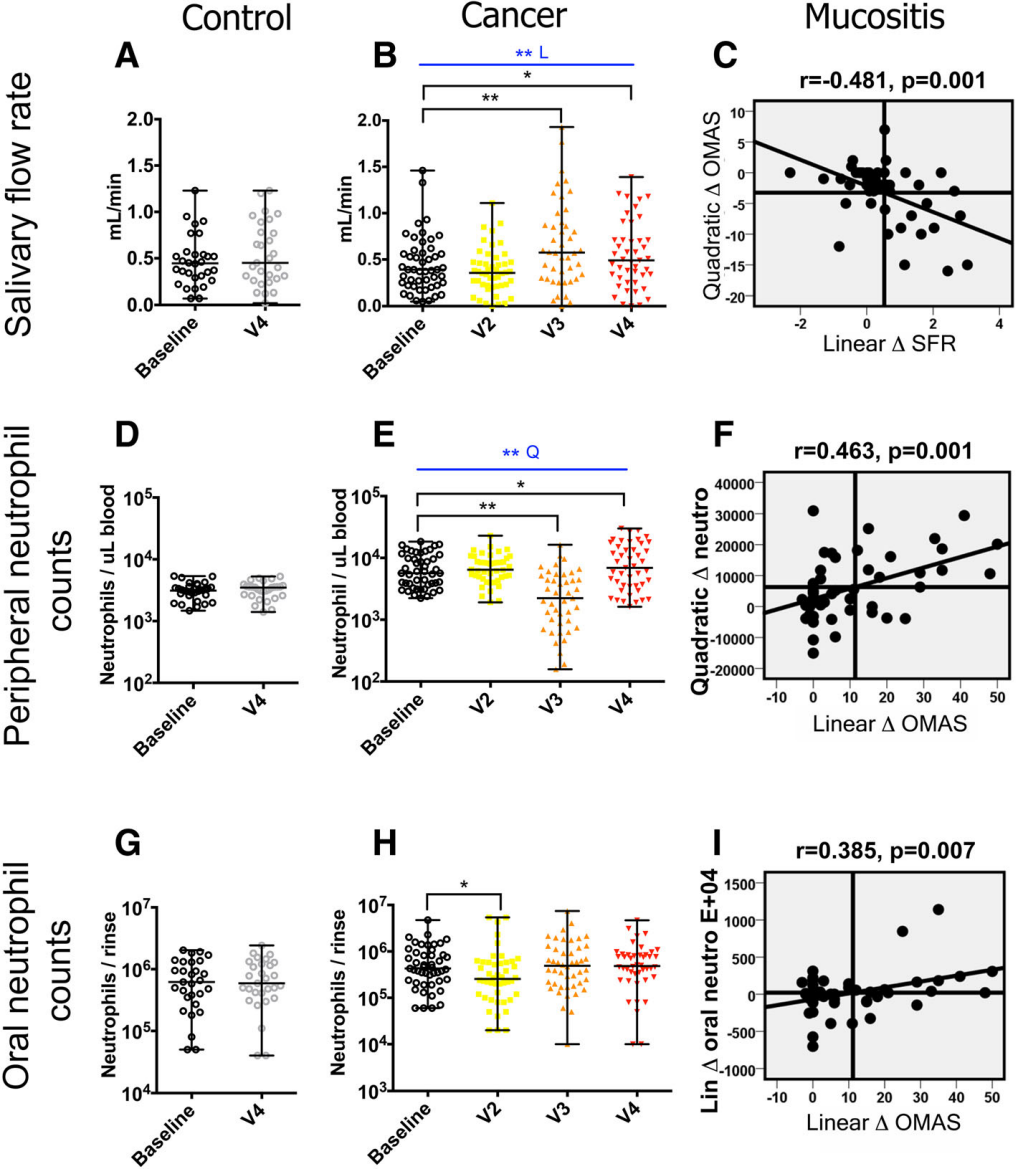
Changes in salivary flow rate and peripheral and oral neutrophils during chemotherapy and correlation of these changes with oral mucositis severity. **a**, **b** Salivary flow rate (SFR) in control and cancer subjects. A statistically significant increase in SFR was seen in cancer subjects at V3 and V4 compared to baseline. Also, the linear change (L) in SFR during chemotherapy was significant. **c** A correlation between the negative quadratic change in OMAS (low, high, low) and the positive linear change in SFR in cancer subjects indicating SFR increased concomitant to or following mucositis. Each data point in the plot represents the change in a subject and was generated by transforming data from each visit according to orthogonal polynomial contrast coefficients followed by aggregation of the data from the four visits. **d**, **e** The change in peripheral neutrophils in control and cancer subjects. A statistically significant decrease during chemotherapy was seen at V3 and V4 compared to baseline. Also, the change during chemotherapy modelled with a quadratic polynomial contrast (*Q*) was significant. **f** A correlation between the positive quadratic change in peripheral neutrophils (high, low, high) and the positive linear change in OMAS indicating a correlation between neutrophil depletion and mucositis severity. **g**, **h** The change in oral neutrophils in control and cancer subjects. A statistically significant decrease was seen at V2 during chemotherapy. **i** A positive correlation between the linear change in oral neutrophils and mucositis severity. * indicates a *p* value < 0.05 and ** < 0.01

A longitudinal analysis was next conducted to better understand if disruptions in two important oral homeostatic mechanisms, saliva and neutrophil surveillance, occurred during chemotherapy and were associated with mucositis. Changes in these variables were evaluated by comparing values at each visit to baseline and by modelling the change over four visits with linear or quadratic polynomial contrasts. In contrast to previous reports [15], we found that SFR increased during chemotherapy (Fig. 2b, *p* = 0.06 at V3; *p* = 0.028 at V4; and *p* = 0.002 for linear change). Increased SFR correlated with more severe mucositis, as shown by significant correlations between the linear increase in SFR during the cycle and the change in mucositis symptoms modelled either quadratically (Fig. 2c, *p* = 0.001) or linearly (Additional file 2: Table S3, *p* = 0.003). These findings suggested mucositis was not associated with decreased saliva, but instead, mucosal inflammation and ulceration occurred concomitant with and/or were followed by increased SFR.

Since neutrophil surveillance could affect oral homeostasis by controlling the growth of oral commensals, which could in turn affect mucosal health, we evaluated the effect of chemotherapy on blood and oral neutrophil counts. As expected due to antineoplastic cytotoxicity, blood neutrophil counts in the cancer group decreased at V3 in comparison to baseline levels (*p* = 0.00009); however, at V4 neutrophil counts rebounded (Fig. 2e). Decreased blood neutrophils in the middle of the cycle (positive quadratic change) correlated with a linear increase in mucositis severity (Fig. 2f, *p* = 0.001 and Additional file 2: Table S3, *p* = 0.001). Oral neutrophils enter the oral cavity through the thin junctional epithelium at the gingiva/tooth interface and via transmucosal migration [21]. Although chemotherapy decreased blood neutrophils, oral granulocytes did not follow a similar pattern, only decreasing slightly at V2 (Fig. 2h, *p* = 0.015). A linear change in oral granulocytes positively correlated with a linear change in mucositis severity (Fig. 2i, *p* = 0.007). Taken together, these findings indicate that despite decreased blood neutrophils as a consequence of chemotherapy, these cells were still being recruited into the oral tissues, with mucositis lesions promoting increased efflux of granulocytes into the oral space.

Additional file 2: Table S3 shows other demographic and clinical characteristics that correlated with the longitudinal clinical progression of oral mucositis. The negative quadratic change in mucositis severity (low, high, low) was associated with 5-FU (*p* = 0.004) but also with number of prosthetic teeth (*p* = 0.003) and wearing a removable oral prosthesis (*p* = 0.002). A linear change in mucositis severity, indicating subjects with more severe mucositis late in the cycle (delayed healing), positively correlated with smoking (*p* = 0.007), steroid intake (*p* = 0.002), and cisplatin dose (*p* = 0.002), while it negatively correlated with doxorubicin dose (*p* = 0.00001).

### Disruption of salivary bacterial communities during chemotherapy correlates with mucositis severity

**Fig. 3:**
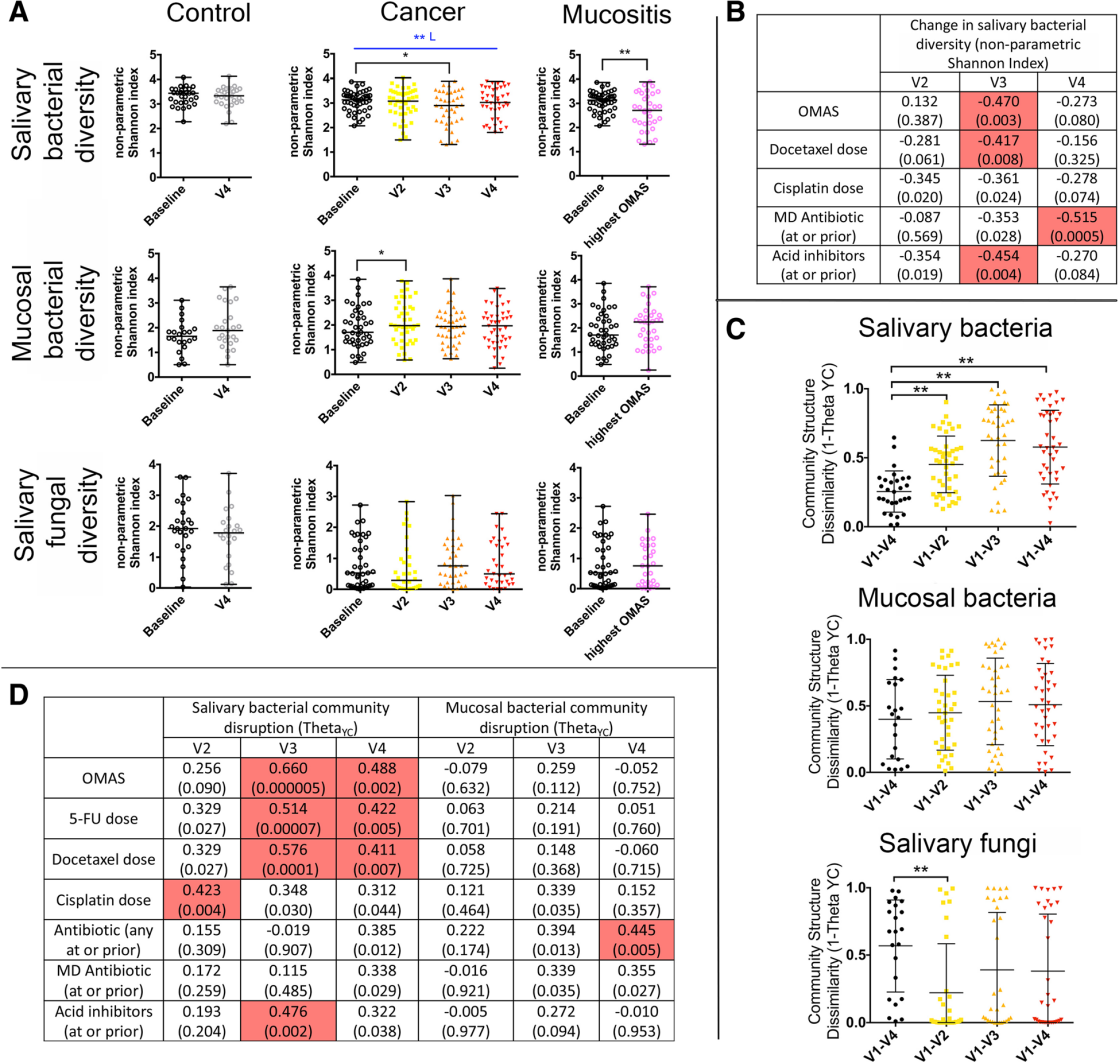
Changes in the oral microbiome during chemotherapy and in relation to the development of oral mucositis. **a** Bacterial and fungal microbiome diversity in control and cancer subjects. Salivary bacterial diversity decreased at visit 3 compared to baseline and also the linear change (L) in diversity was significant. Decreased salivary bacterial diversity was also seen at the visit with the highest OMAS. Mucosal bacterial diversity increased at V2, while no changes were seen in salivary fungal communities. **b** Variables significantly correlated with the change in salivary bacterial diversity at each visit when compared to baseline. Data represent Spearman correlation coefficients with *p* values in parenthesis. Colored cells show correlations significant after adjustment for multiple comparisons via the FDR method. **c** Changes in community structure as measured by the Theta_YC_ distance from baseline (V1) to each visit. Black data points indicate changes in control subjects and color data points indicate cancer subjects. ** indicates a *p* value < 0.01 when comparing to control subjects via Mann-Whitney Rank tests. **d** Variables significantly correlated with changes in salivary and mucosal bacterial community structure (1-Theta_YC_ distance from baseline to each visit). Data represent Spearman correlation coefficients with *p* values in parenthesis. Colored cells show correlations significant after adjustment for multiple comparisons via the FDR method

The oral microbiome was longitudinally evaluated by sequencing of salivary and mucosal 16S rRNA gene amplicons and salivary internal transcribed spacer (ITS)-1 DNA amplicons. Chemotherapy disrupted the oral microbiome with the most severe changes seen in salivary bacterial communities. Salivary bacterial alpha-diversity decreased during the cycle (*p* = 0.01 at V3 and *p* = 0.009 for linear change) and at the visit with the most severe mucositis (Fig. 3a, *p* = 0.001). Mucosal bacterial community diversity was only minimally affected, with a small increase seen at V2 (*p* = 0.042), while salivary fungal community diversity did not change during chemotherapy (Fig. 3a). Changes over time in salivary bacterial diversity modelled with orthogonal polynomial contrasts showed a correlation of the linear change with the dose of 5-FU and intake of a multi-dose antibiotic, and of the quadratic change with docetaxel and cisplatin doses (Additional file 2: Table S3). To better understand the contributions of different predictors to changes in salivary bacterial diversity and evaluate their effect at each time point during the cycle, we also conducted a by visit analysis, calculating the change in diversity from each visit to baseline (Fig. 3b). This analysis showed that at V2, there was a trend for a correlation between decreased salivary bacterial diversity with higher cisplatin dose and intake of acid inhibitors. At V3, decreased diversity correlated with higher OMAS scores at the same visit (*p* = 0.003), a higher docetaxel dose (*p* = 0.008), and use of acid inhibitors (*p* = 0.004). The use of a multi-dose antibiotic and the dose of cisplatin showed a trend for a negative correlation with diversity. At V4, the use of a multi-dose antibiotic (*p* = 0.0005) was associated with a decrease in diversity. A linear regression analysis was run considering the change in diversity at V3 as dependent variable and OMAS score, the use of multi-dose antibiotic and use of acid inhibitors as predictors. This analysis showed that OMAS (*β* = − 0.300, *p* = 0.046) and acid inhibitor use (*β* = − 0.301, *p* = 0.045), but not antibiotic use, remained significantly associated with the change in diversity at V3. In summary, different factors appeared to have influenced salivary bacterial alpha-diversity with changes at V3 associated with mucositis severity and acid inhibitor use and at V4 with the intake of a multi-dose antibiotic.

Echoing the changes seen in alpha-diversity, salivary bacterial community structure (beta-diversity) was also disturbed during chemotherapy (Fig. 3c). Mucosal bacterial communities followed a similar trend as their salivary counterparts but their changes were not greater than those of non-cancer controls. The structure of salivary fungal communities was not disturbed by chemotherapy (Fig. 3c).

To inquire which patient factors were associated with disruptions in community structure, the Theta_YC_ distances between baseline to each visit, indicating the magnitude of change, were evaluated for a correlation with clinical characteristics (Fig. 3d). This analysis showed that the degree of salivary bacterial community disruption at V2 correlated with cisplatin dose (*p* = 0.004). At V3, salivary bacterial Theta_YC_ distances positively correlated with the degree of mucositis severity (*p* = 0.000005), with the doses of 5-FU (*p* = 0.00007) and docetaxel (*p* = 0.0001) and with the use of acid inhibitors (*p* = 0.002). Linear regression using salivary bacterial Theta_YC_ distances at V3 as the dependent variable and OMAS scores and acid inhibitor use as predictors showed both variables remained independently associated with community structure change (OMAS *β* = 0.527, *p* = 0.0002 and acid inhibitor *β* = 0.287, *p* = 0.033). At V4, salivary bacterial community disruption correlated with mucositis severity (*p* = 0.002) and with 5-FU (*p* = 0.005) and docetaxel (*p* = 0.007) doses. Surprisingly, antibiotic use only showed a trend for a correlation with salivary bacterial community disruption, and this occurred only at V4 (*p* values below 0.05 but not significant after adjustment for multiple testing). A linear regression analysis evaluating the effect of OMAS, multi-dose antibiotics, and acid inhibitors on Theta_YC_ distances at V4 showed that when the effect of each of the three predictors was adjusted for the effect of the others, OMAS (*β* = 0.347, *p* = 0.014) and antibiotics (*β* = 0.281, *p* = 0.048) remained significant below a 0.05 p level. For mucosal communities, the only significant correlation seen was between Theta_YC_ distances at V4 and antibiotic use. A correlation trend was seen between mucosal Theta_YC_ distances at V3 with cisplatin dose and antibiotics. Overall, these results show that mucositis severity and acid inhibitor use were the main predictors associated with salivary bacterial community structure disruption during the cycle. Antineoplastic doses were also associated with Theta_YC_ distances, but it should be noted that their independent association could not be evaluated as they are highly correlated with OMAS scores. Antibiotics seemed to only affect the microbiome at V4 having a greater effect on mucosal communities than on their salivary counterparts.

We next asked if disruption of salivary bacterial community structure occurred in a consistent direction among subjects as visualized by PCoA plots (Additional file 1: Figure S2). At V2, there was no significant separation of samples from baseline communities. At V3 and V4, there was a significant separation of the corresponding data clouds from baseline samples, although not all microbiomes changed in a uniform direction (Additional file 1: Figure S2A–B, *p* = 0.006 at V3 and *p* = 0.003 at V4). Although microbiome changes were heterogeneous among subjects, microbiome variability along axis 2 in both the V3 and V4 plots (Additional file 1: Figure S2A–B) correlated with OMAS score, doses of 5-FU, and docetaxel, with receiving a multi-dose antibiotic prior to V3, and with changes in alpha-diversity.

Changes in the proportions of individual taxa during chemotherapy were also evaluated. Additional file 1: Figure S2C shows the number of taxa that significantly changed at each visit, with the greatest number of altered taxa seen in salivary bacterial communities at V3 and V4. Additional file 1: Figure S3 and Additional file 3: Table S4 show the identity of these taxa. At V3, all changes in saliva were due to significant decreases in relative abundance. Several Gram-negative species from the genera *Prevotella*, *Selenomonas*, *Leptotrichia*, *Tannerella*, *Campylobacter*, *Capnocytophaga*, and *Parvimonas* showed a trend to increase in relative abundance at V3, but no species reached significance, highlighting the heterogeneity among individuals in the species that replaced those that decreased. At V4, we observed both significant increases and decreases in abundance.

Taken together, these results show that the salivary bacterial microbiome was profoundly altered during chemotherapy. While intake of multi-dose antibiotics and acid inhibitor use during the cycle were associated with some of the observed microbiome modifications, disruption of salivary bacterial communities was also independently associated with oral mucositis severity. The total doses of 5-FU, docetaxel, and cisplatin, associated with more severe mucositis, also correlated with greater salivary bacteriome disruption.

### Oral mucositis is associated with oral bacteriome dysbiosis

**Fig. 4:**
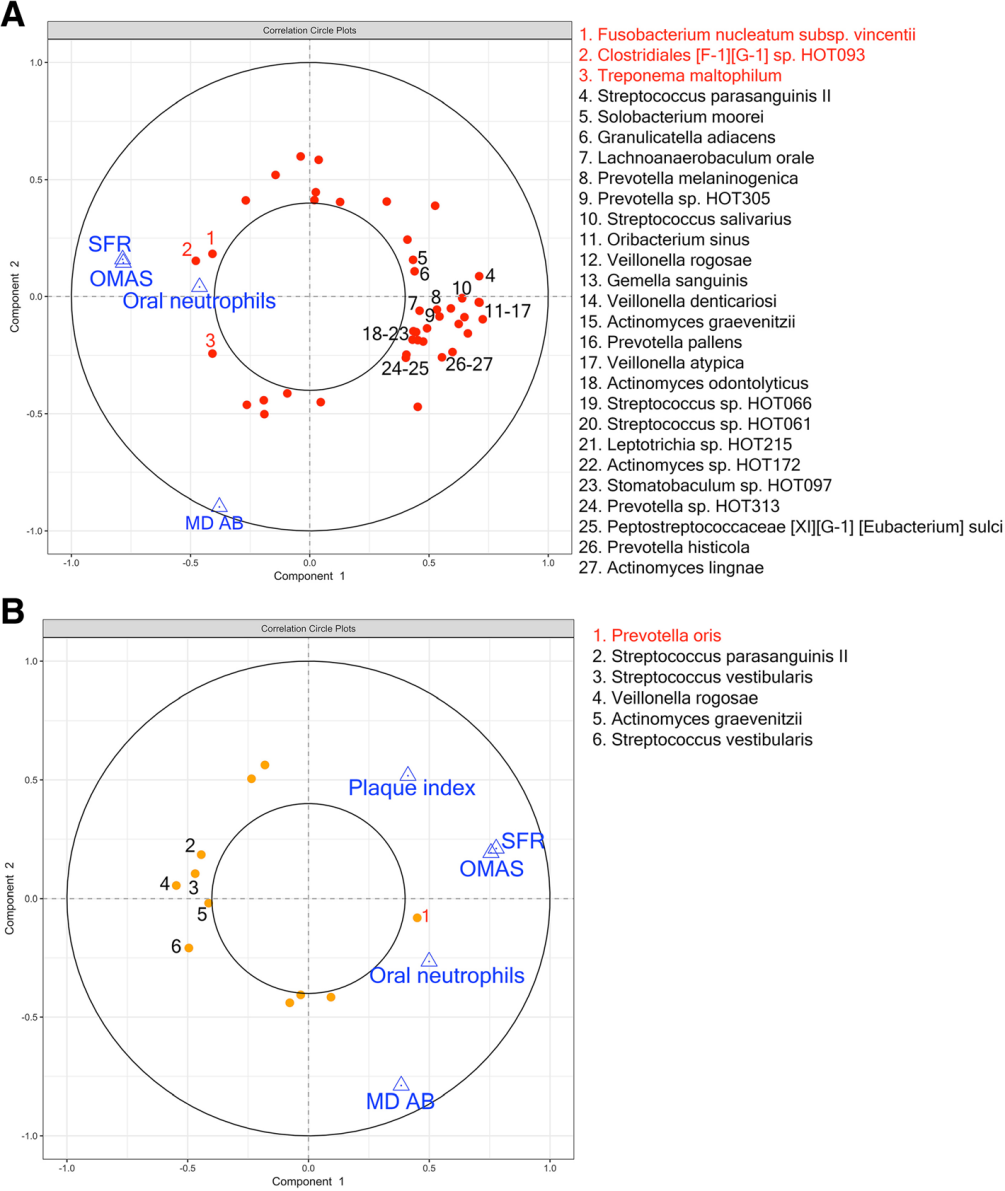
Longitudinal covariation of bacterial relative abundances and clinical signs of oral mucositis during chemotherapy. **a** A circle correlation plot depicting covariation of salivary bacterial abundances with oral mucositis severity (OMAS) and other significant clinical variables, as determined via multi-level sPLS analysis. Bacterial species appear as red circles and clinical variables are shown as blue triangles. Data points were placed in the plot according to their correlation with the two main components. Positively correlated variables follow the same direction from the origin. The greater the distance from the origin, the stronger the association. Only variables with a correlation coefficient greater than 0.4 are shown. Red numbers and corresponding names indicate bacterial species positively correlated with OMAS (enriched as mucositis severity increased), while black numbers and names indicate bacterial species negatively correlated with OMAS (depleted during severe mucositis). SFR salivary flow rate, MD AB multi-dose antibiotic intake; **b** a similar analysis done for mucosal bacterial taxa. Bacterial species appear as orange circles and clinical variables as blue triangles. Red numbers and names indicate species positively correlated with OMAS, while black numbers and names indicate bacterial species negatively correlated with OMAS

To evaluate the longitudinal covariation of microbial taxa and clinical variables during chemotherapy, we conducted a multilevel multivariate sparse partial least square analysis focusing on bacterial species enriched or depleted in accordance with mucositis severity. Figure 4a shows the correlations between the longitudinal change in salivary bacterial proportions and clinical variables, including OMAS scores. Mucositis severity positively correlated with enrichment of 3 salivary Gram-negative species (taxa 1 to 3 in Fig. 4a), namely *Fusobacterium nucleatum* subsp. *vincentii*, an uncultured *Clostridiales*, and *Treponema maltophilum*, all taxa associated with other oral inflammatory conditions [19]. Mucositis severity also correlated with a decrease in proportions of 24 salivary bacterial species (taxa 4 to 27 in Fig. 4a), among them abundant commensals from genera typically associated with oral health such as *Streptococcus*, *Actinomyces*, *Gemella*, *Granulicatella*, and *Veillonella* [19, 22]. Consistent with previous analyses (Fig. 2), mucositis severity positively correlated with SFR and oral neutrophils (Fig. 4a). It should also be noted that the intake of a multi-dose antibiotic did not correlate with changes in the same salivary bacteria associated with OMAS. This result further confirmed antibiotic treatment was not the cause of mucositis-associated dysbiosis.

A similar analysis of mucosal bacterial communities found that changes in only a few taxa correlated with mucositis severity (Fig. 4b). Changes, however, were consistent with those seen in saliva, with enrichment of the Gram-negative, inflammation-associated *Prevotella oris* [19] and lower abundance of *Streptococcus*, *Veillonella*, and *Actinomyces* species as OMAS scores increased (Fig. 4b).

Taken together, these results revealed that mucositis is associated with depletion of prevalent health-associated commensals (likely symbionts) and enrichment of Gram-negative species typically enriched during oral inflammation (likely pathobionts). These changes are reminiscent of dysbiotic shifts seen in other oral inflammatory conditions such as gingivitis and periodontitis [19], although the changes are not directly related to these conditions as mucositis did not correlate with the presence of severe periodontitis.

### The antimicrobial activity of antineoplastics is unlikely to explain chemotherapy-associated dysbiosis

We found that the change in relative abundance of many species associated with mucositis (in Fig. 4a, b) also correlated with the use and dose of specific antineoplastics (Additional file 1: Figure S4A). For instance, depletion of *Streptococcus salivarius* during mucositis correlated with higher doses of 5-FU and docetaxel (*p* = 0.004 in both cases). Additional file 1: Figures S4B and S4C show taxa depleted during mucositis in subjects taking 5-FU, but that did not change in subjects on doxorubicin who also presented with mucositis, albeit of less severity. These results raised the possibility that the antimicrobial activity of specific antineoplastics was contributing to the selective depletion or enrichment of oral bacteria during chemotherapy-induced mucositis. Indeed, when we exposed salivary communities to physiological concentrations of 5-FU or 5-FU and docetaxel, we observed a decrease in viability of the total culturable community after a 2-h incubation suggesting these drugs have the potential to modify the microbiome (Additional file 1: Figure S4D). We hypothesized that if 5-FU was responsible for the dysbiotic changes seen during chemotherapy, in particular those shifts associated with mucositis, then depleted taxa should be susceptible to the drug while enriched species would be resistant. To test this, bacterial strains of the depleted *S. salivarius*, *Streptococcus parasanguinis*, *Veillonella atypica*, and *Veillonella rogosae* and the enriched *Fusobacterium nucleatum* were exposed to 5-FU. After a 2-h incubation, however, only *Veillonella atypica* showed decreased viability at the same physiologic concentration (7.7 μM) tested with whole saliva (Additional file 1: Figure S4E). In fact, many of the commensals depleted during mucositis were resistant to killing by 5-FU even at drug concentrations likely to be above the physiologic range (e.g., 770 μM). The ability of 5-FU to inhibit the growth of *S. salivarius* and *F. nucleatum* was also tested with these evaluations showing that even high concentrations of 5-FU did not completely inhibit *S. salivarius*, while the growth of *F. nucleatum* was inhibited by all concentrations tested (Additional file 1: Figure S4F–G). Since taxa negatively correlated with 5-FU and mucositis were not consistently sensitive to 5-FU exposure and a species enriched during mucositis was not resistant to the drug, we conclude that an antimicrobial effect of 5-FU is unlikely to explain the microbiome dysbiosis associated with mucositis. Chemotherapeutics, however, could still temporarily modify the oral microbiome, especially while salivary drug concentrations are in the high range, but these changes do not seem to be associated with mucositis-specific shifts.

### Mucosal lesions did not differ in their microbiome from healthy mucosa

The analysis of mucosal communities used combined libraries from sites with different clinical appearance (healthy, erythematous, ulcerated) obtained from the same subject. An analysis using separate libraries (see list of libraries in Additional file 2: Table S5) was also conducted to explore whether mucosal lesions harbored a microbiome distinct from their subject-matched healthy site counterparts. We did not observe differences in either alpha-diversity (Additional file 2: Table S6) or in the abundance of individual taxa when comparing visit-matched, within-subject, mucosal communities with different clinical appearance (healthy vs erythematous or healthy vs ulcerated). This result indicates that mucosal surface changes were not selecting for a specific microbiome colonization at the lesion sites but rather mucositis-associated dysbiosis was affecting the mouth as a whole.

### The oral epithelium responds to chemotherapy by upregulating genes involved in innate immune responses and apoptosis

**Fig. 5.**
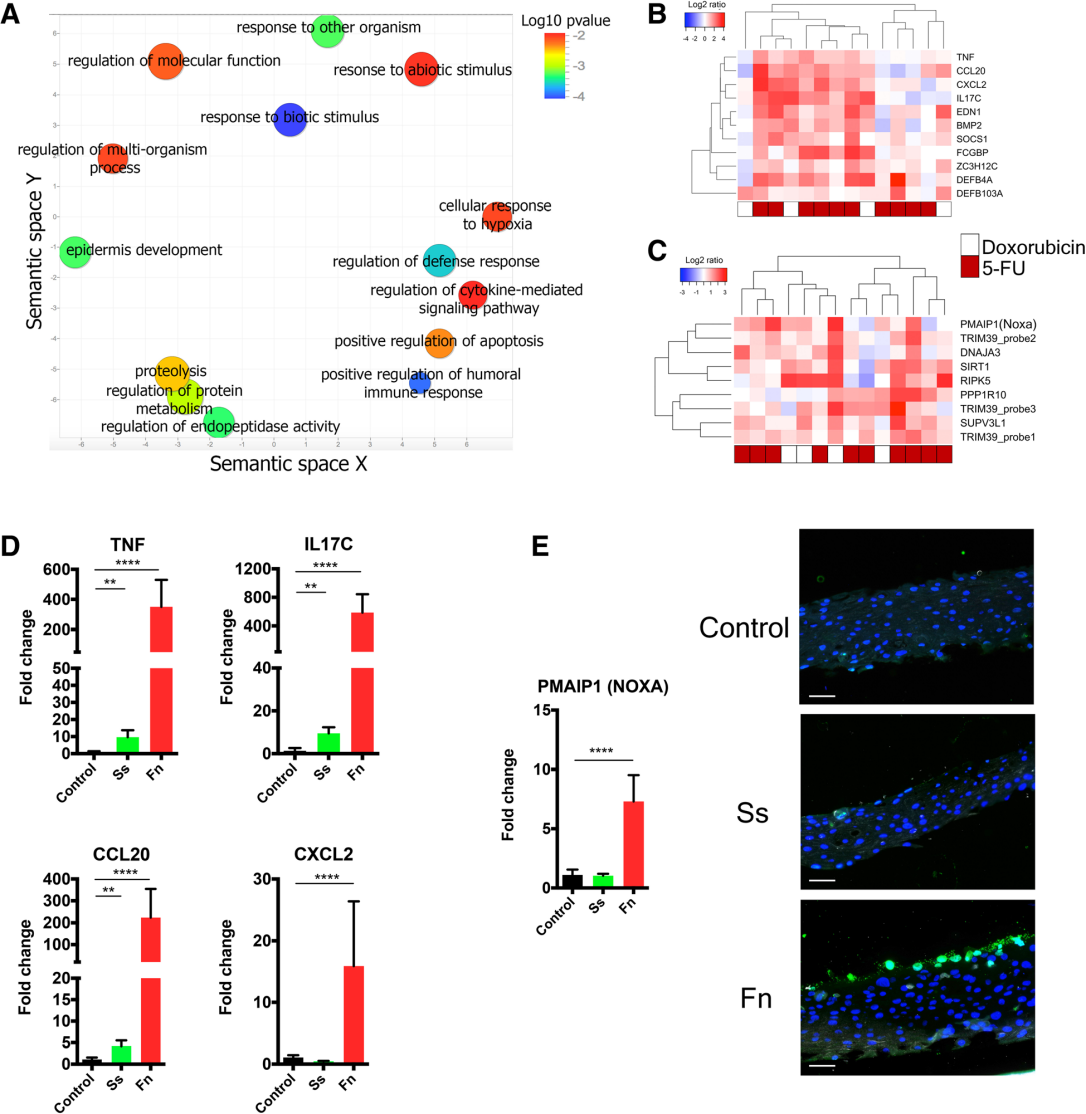
Epithelial responses to chemotherapeutic treatment and potential for commensals to modulate mucosal response. Changes in the oral epithelial transcriptome during chemotherapy (baseline to V3) were evaluated in 14 subjects via DASL-whole genome arrays. **a** A scatter plot showing significantly upregulated gene ontology (GO) terms summarized using REVIGO. Size of each circle represents GO term frequency (log scale) and color its *p* value (log scale), with lower *p* values in blue. **b** genes related to the immune response that were upregulated more than twofold during chemotherapy. **c** Genes related to apoptosis upregulated more than twofold during chemotherapy. **d** The expression of selected immune genes as evaluated via real-time PCR after exposure of a 3D multilayer oral epithelial construct to *Streptococcus salivarius* ATCC 9222 (Ss) or *Fusobacterium nucleatum* subsp. *vincentii* ATCC 49256 (Fn). **e** Expression of the proapoptotic gene *PMAIP1* (NOXA) as measured via real-time PCR and micrographs depicting multilayer oral epithelial constructs stained with a fluorescent TUNEL assay to evaluate cell death. TUNEL-positive cells appear green and nuclei in blue. Bar = 50 μM

Since little information exists on genes and pathways mediating oral mucosal injury, we conducted a gene expression analysis in a subset of 14 subjects comparing oral epithelial transcript levels between baseline and V3. The clinical characteristics of the subjects analyzed are shown in Additional file 2: Table S7. SAM analysis showed 158 upregulated and two downregulated genes at V3 (> 1.5-fold) (Additional file 4: Table S8). Although there was substantial variability in the response of subjects, this was not related to the type of chemotherapeutic regimens subjects were taking according to clustering analysis. Figure 5a shows a summary of upregulated gene ontology (GO) term categories (for a complete list see Additional file 5: Table S9), which included genes involved in epidermis development, apoptosis and cell death, proteolysis, immune responses, and response to stress. Figure 5b shows the main genes involved in innate immune responses that were upregulated. These included *TNF*, which is a key inflammatory mediator that leads to activation of nuclear factor κB (NF-κB) but could also activate apoptosis via the extrinsic pathway or induce necroptosis [23]. Other genes involved in epithelial innate immune responses to microbial commensals such as *IL17C*, *CCL20*, *CXCL2*, *DEFB4A*, and *DEFB103A* were also upregulated suggesting a microbiome influence on mucosal responses during chemotherapy. Another category of genes upregulated that could have a role in mediating mucosal damage was genes related to apoptosis (Fig. 5c). Of particular interest was the upregulation of *PMAIP1*, the gene encoding the proapoptotic Bcl2 homology 3 (BH3)-only protein NOXA, which activates the intrinsic apoptosis pathway [24], and has been shown to mediate cisplatin-induced cell death [25].

### Potential for bacterial dysbiotic changes to affect epithelial responses

We next explored the potential for dysbiotic microbiome changes associated with mucositis severity to affect epithelial responses. As Fig. 5d shows, exposure of a 3D multilayer oral epithelial construct to the potential symbiont *S. salivarius*, depleted during mucositis, provoked minimal induction of innate immune response genes. In contrast, challenge of epithelial tissues with a similar load of the potential pathobiont *F. nucleatum*, associated with more severe mucositis, produced a dramatic upregulation of *TNF*, *IL17C*, *CCL20*, and *CXCL2*. We also observed that *F. nucleatum* induced an upregulation of *PMAIP1* and, consistent with this effect, tissues displayed death of cells in the apical layer (Fig. 5e). These results suggest that dysbiosis triggered by chemotherapy could affect epithelial responses and may play a role in the course of oral mucositis lesions.

## Discussion

Despite the adverse clinical impact of oral mucositis, there is insufficient knowledge on its pathophysiology. Enhanced understanding of events that mediate oral mucosal damage and whether microbial signaling modifies the response to antineoplastics is important for the future development of novel therapies. To our knowledge, our work represents the most comprehensive characterization to date of changes in the human oral environment during chemotherapy in relation to oral mucositis. Our study cohort included patients undergoing treatment based on either 5-FU or doxorubicin. Although both groups developed oral mucositis, its incidence was higher in the 5-FU group with a highly significant correlation seen between the total dose of 5-FU and the severity of lesions. 5-FU is an anti-metabolite drug widely used either alone or as a foundational therapeutic in combination treatment regimens for a range of cancers, including colorectal, breast, and cancers of the aerodigestive tract [7]. A recently developed animal model showed that 5-FU induces atrophy of the oral mucosa with reduced proliferation, increased cell death, upregulation of pro-inflammatory mediators, and compromised barrier integrity [26]. The mechanisms by which 5-FU alone or in combination with adjunct chemotherapeutics exerts its toxic effects on the human oral mucosa, however, remain incompletely understood, with the role of the microbiome in oral mucositis almost entirely unexplored.

Our work demonstrates that the oral microbiome is disrupted by chemotherapy. Although we expected chemotherapy to affect the oral mycobiome due to myelosuppression, we did not observe major changes in the composition of fungal communities. Instead, it was the oral bacteriome that displayed major modifications. These changes were of greater magnitude in salivary communities, but were also detected in mucosal samples. Shifts in the oral bacteriome were strongly correlated with mucositis severity. Bacteriome shifts, however, affected the mouth as a whole and were not specific to the surfaces of lesions as we did not detect any differences in the mucosal bacterial communities present at healthy, erythematous, or ulcerated sites. It should be noted, however, that since swabs were used to sample mucosal lesions, there could have been subsurface microorganisms in lesions not captured by our sampling approach.

An exploration of possible causes of bacteriome shifts found that the use of a multiple dose antibiotic correlated with decreased salivary bacterial diversity but showed only a weak correlation with changes in salivary bacterial community structure. Antibiotic intake mostly affected the oral microbiome at the last visit (V4) and its effect was independent from mucositis-associated dysbiotic shifts. Furthermore, a single-dose prophylactic antibiotic exposure did not correlate with oral microbiome changes. This lack of a major effect of antibiotics on the oral microbiome is not surprising, as previous studies indicate the oral microbiome is resistant to change after systemic antibiotic administration, in contrast to gut communities, which are easily disrupted by antibiotics [27]. Microbiome changes during chemotherapy and mucositis could be neither explained by impaired salivary flow, which in fact was increased during chemotherapy and positively associated with mucositis severity. This result contrasted with previous reports in which patients undergoing repeated chemotherapy had decreased SFR [15]. The discrepancy with our results is probably due to the fact that our cohort was comprised of patients who were either chemotherapy-naïve or in their first cycles of treatment. It is possible that repeated exposure to antineoplastics could cause induction of temporary glandular damage therefore affecting SFR. Similarly, dysbiotic microbiome shifts were not related to decreased oral granulocyte availability, which in fact was positively correlated with more severe mucositis. Increased oral neutrophils during mucositis may be the result of upregulation in chemoattractants such as CXCL2 by the epithelium, as seen in our transcriptome data, in combination with barrier defects through which neutrophils escape tissues. Furthermore, evaluation of a direct antibacterial effect of chemotherapeutic drugs did not support the possibility that chemotherapeutics, in particular 5-FU, were responsible for microbiome shifts associated with mucositis, although 5-FU showed antimicrobial activity against whole salivary communities and against a few of the species tested. Salivary concentrations of 5-FU have been measured at approximately 77 μM 12 min after the start of 5-FU administration, but concentrations rapidly decrease to about 7 μM after 3 h and become undetectable minutes after the end of a 22-h infusion [28].Based on our results, it is thus possible that for a short period of time during 5-FU administration, the viability of selected oral bacteria is affected by the drug, but salivary 5-FU concentrations rapidly decline allowing oral commensals to recover. In addition, it should be noted that most study subjects received 5-FU within a maximum 5-day window, while most of the microbiome changes associated with mucositis were seen to occur at visits 3 and 4, which were days apart from the last infusion. Based on the salivary drug kinetics described above, it is therefore unlikely that mucositis-associated microbiome shifts were due to a direct antibacterial effect of 5-FU. In support of this, we did not observe that microorganisms whose depletion correlated with high 5-FU doses and more severe mucositis were consistently sensitive to 5-FU, while the growth of *F. nucleatum*, a microorganism enriched during mucositis, was inhibited by the drug.

The correlation of microbiome shifts and mucositis is in agreement with a previous study that evaluated changes in the oral mucosal bacterial communities in children undergoing chemotherapy, finding greater microbiome modifications in children that developed oral mucositis [29]. This prior study, however, did not define specific species associated with lesion development. We found that shifts in bacterial communities during mucositis consist of depletion of abundant species from genera associated with health and enrichment of Gram-negative species linked to other oral inflammatory conditions [19]. Mucositis-associated microbiome shifts may have resulted from the release of inflammatory products into the oral environment after chemotherapy-induced damage and inflammation. Bacterial species from genera found enriched in mucositis are known to thrive using inflammatory products as nutrients [30, 31], and therefore, the observed mucositis-associated microbiome shifts could have resulted from alterations in oral nutritional resources that promote the growth of these Gram-negative species.

To better understand epithelial cellular events altered by chemotherapy and potential contributors to oral mucosal injury, we performed a global gene expression analysis of changes in the oral epithelium from baseline to V3. Although the specific sites sampled were not affected by mucositis, most subjects presented with lesions in other areas of the mouth at the time of sampling. One upregulated category was that of genes involved in the innate immune response, including here *TNF*, *IL17C*, *CXCL2*, and *CCL20*, which are cytokines typically induced in epithelial tissues by stimulation with microorganisms, but not part of the response of cell lines after an antineoplastic challenge [32–36]. This suggested that some of the responses seen in vivo were the result of microbial-host cross talk. In vitro challenge of a 3D oral epithelial construct with *F. nucleatum*, a microorganism associated with more severe mucositis, confirmed the potential for a dysbiotic microbiome to contribute to upregulation of these innate immune response genes during chemotherapy. In contrast, *S. salivarius*, a microorganism depleted during mucositis, only promoted a slight upregulation or had no effect on innate immunity mediators. The role microbially induced innate immune responses could play in amplifying oral epithelial damage during chemotherapy warrants further investigation.

An expected category of upregulated genes in the oral epithelium during chemotherapy was that of apoptosis-related genes. Among them, the pro-apoptotic gene *PMAIP1* (NOXA) is known to mediate antineoplastic-induced cytotoxicity [25]. NOXA is a BH3s death sentinel that acts by directly inducing stepwise, bimodal activation of BAX–BAK, which mediate permeabilization of mitochondria, followed by the release of cytochrome c and caspase activation [24]. NOXA also binds to the anti-apoptotic protein MCL-1 and may induce apoptosis by preventing its activity [24, 37, 38]. *PMAIP1* is upregulated in gastric epithelial cells upon *Helicobacter pylori* infection, which suggests a link between microbial sensing and *PMAIP1* transcription [39]. Unexpectedly, we also observed that in vitro challenge of oral epithelial cells with *F. nucleatum* upregulated expression of *PMAIP1* and consistent with this, *F. nucleatum*-challenged tissues displayed cell death markers. This suggested that the upregulation of signals leading to cellular death could also occur in response to oral dysbiosis, which may serve to aggravate epithelial injury.

Another factor identified as associated with mucositis was wearing a removable prosthesis. Contact of a denture with oral tissues may constitute a mechanical insult that aggravates antineoplastic-driven epithelial injury. Dentures are also known to promote the overgrowth of oral commensals, which in turn could modify mucosal damage [40]. Additional factors associated with a linear increase in mucositis severity were smoking and steroid use during the cycle. Both smoking and steroids are likely to modify innate immune responses of mucosal tissues that are important for controlling dysbiosis [41, 42], and may play a role in mucositis pathophysiology.

## Conclusions

In summary, our work shows that the severity of oral mucositis lesions correlates with the degree of oral microbiome dysbiosis, with enrichment of potential Gram-negative pathobionts and depletion of health-associated symbionts in severe mucositis. These microbiome alterations resemble the dysbiotic shifts associated with other oral diseases in which changes in the environment and nutrient resources, due to inflammation, induce the outgrowth of low abundant species, which in turn dysregulate host defenses leading to damage [20]. Indeed, *F. nucleatum*, a potential pathobiont enriched as mucositis became more severe, showed an ability to induce pro-inflammatory and apoptotic responses in a 3D oral epithelium model, while *S. salivarius*, a potential symbiont depleted in severe mucositis, was tolerated. While antineoplastics represent the primary initiators of mucositis, our observations also suggest that antineoplastic-triggered inflammation may induce microbiome disruptions and that such dysbiotic microbiome could play a role in the clinical course of lesions aggravating epithelial injury. Further studies are warranted to elucidate whether interfering with dysbiotic events could reduce oral mucositis incidence and/or severity.

## Methods

### Study design

This observational prospective study with two cohorts was approved by the Institutional Review Board at UConn Health (IRB number IE-11-037 J-2) and conformed with STROBE guidelines. Written informed consent was received from all participants. Additional file 1: Figure S1 shows the study design and inclusion and exclusion criteria. Subjects planned to or already receiving chemotherapeutic treatment for a solid tumor either on a 5-fluorouracil (5-FU)- or a doxorubicin-based regimen were recruited at the Neag Comprehensive Cancer Center of UConn Health and at the Helen and Harry Gray Cancer Center at Hartford Hospital. Every effort was made to recruit chemotherapy-naïve subjects. From the 49 enrollments in the cancer cohort, 32 were chemotherapy-naïve while 17 had already completed at least one previous cycle. Thirty non-cancer controls were enrolled from the general local population through the UConn Health Dental Clinical Research Center. Cancer subjects were seen at four visits which included a baseline visit completed prior to subjects commencing chemotherapy, and three more visits (V2–V4) within 14 ± 2 days after commencing treatment. Control subjects were seen at two visits, including a baseline and a visit within 14 ± 2 days (V4).

### Collection of demographic and medical data and oral evaluation

Medical information was obtained via questionnaires and from medical charts. All subjects received at baseline an oral evaluation including assessment of the presence of periodontitis via the Community Periodontal Index of Treatment Needs (CPITN) [43], presence and type of prosthetic restorations, number of teeth, and presence of visible cavitated carious lesions. In addition, at all visits subjects were evaluated for the presence and severity of mucositis per the World Health Organization (WHO) scale, which assesses oral soreness, erythema, ulceration, and ability to eat using a 0–4 scale, giving a single score for the entire oral cavity [44]. A score of 0 indicates no signs or symptoms; 1 indicates erythema and soreness; 2 indicates ulcers, able to eat solids; 3 indicates ulcers, requires liquid diet (due to mucositis); and 4 indicates ulcers, alimentation not possible (due to mucositis). A second mucositis assessment method, the Oral Mucositis Assessment Scale (OMAS) was also used [45]. The OMAS provides an objective assessment of oral mucositis based on an evaluation of the appearance and the extent of redness and ulceration in various areas of the mouth. Nine oral mucosal sites were evaluated for presence and severity of mucositis ulcerative lesions assigning to each site a score from 0 to 3 depending on lesion size, and for severity of erythema, assigning each site a score from 0 to 2. These scores were then aggregated per site and therefore each site could have a score ranging from 0 to 5. Scores from all nine sites were then aggregated for a total mouth score ranging from 0 to 45. Aggregated total mouth scores were used for analysis. At all visits, a subjective evaluation of oral dryness was also conducted [46], and an assessment of the amount of plaque on teeth, as determined via the Plaque Index of Silness and Löe [47].

### Salivary flow rate determination and collection of samples for microbiome evaluation

At each visit, unstimulated saliva and mucosal swabs were collected for microbiome evaluation. Participants were instructed to avoid eating or drinking anything other than water for 1 h prior to each study visit. Unstimulated saliva was collected for 5 min by having subjects lean forward over a sterile funnel attached to a sterile vial placed on ice. Saliva was weighted to determine salivary flow rate, and then aliquoted and centrifuged at 6000×*g* for 10 min. Supernatants were removed and pellets stored at − 80 °C. Mucosal swab samples were collected in subjects presenting mucosal health by swabbing the entire right and left buccal mucosa for 10 s each with a single CatchAll™ swab. In subjects presenting with mucosal lesions, separate swabs were collected from healthy mucosal areas, erythematous tissue, and areas with ulceration. Swabs were immediately swirled in a tube containing 500 μL of TE buffer (20 mM Tris pH 7.0, 2 mM EDTA) pressing against the tube walls to release the swab contents. Samples were then stored at − 80 °C.

### Collection of samples for oral epithelium transcriptome analysis

Oral mucosal cytology samples were collected using a Cytobrush™, which was pressed and rotated against a healthy mucosal area for 60 s. If the procedure lead to mucosal bleeding, the sample was discarded to avoid contamination by cells beyond the epithelium. After collection, the brush was swirled in 1 mL of RNAProtect (*Qiagen*), pressing against the tube walls to release the brush contents. Cells were pelleted at 8000×*g* for 20 min, snap-frozen, and stored in liquid nitrogen.

### Evaluation of blood and oral neutrophil counts

Total counts and percentages of neutrophils were obtained from complete blood counts performed by the hospital laboratory using an automated Beckman Coulter analyzer. Oral cells were collected at all study visits by asking subjects to rinse with 10 mL of a bicarbonate solution for 30 s. Oral rinse samples were centrifuged at 1258×*g* for 15 min and pellets were resuspended in Hanks’ Balanced Salt solution supplemented with 2 μg/ml acridine orange. Granulocytes, most of which are neutrophils, characteristically stained as multilobulated cells with this nuclear stain, were enumerated in a hemacytometer under a fluorescence microscope [48].

### DNA extraction for microbiome evaluation

DNA was extracted separately for bacterial and fungal microbiome evaluation. For bacteria, we followed a previously described procedure using lysozyme and proteinase K treatment and the DNeasy Blood and Tissue kit (Qiagen) [49]. For fungi, the DNA extraction protocol involved bead beating with a matrix containing Lysing Matrix B (MP Biomedicals) and very high density 0.5 mm yttrium-stabilized zirconium oxide (95% ZrO2 + 5% Y2O3) grinding media (YSZ) (Glen Mills Inc, Clifton, NJ), followed by extraction using the FastDNA SPIN KIT (MP Biomedicals), as previously described [50].

### Amplification and sequencing of 16S rRNA gene and ITS-1 DNA

Amplicon libraries of the 16S rRNA gene V1–V2 hypervariable regions were generated in triplicate using fusion primers which included universal primers 8F AGAGTTTGATCMTGGCTCAG or 361R CYIACTGCTGCCTCCCGTAG [51]. PCR conditions have been described previously [49]. For mycobiome characterization, fusion primers containing fungal-specific ITS1F forward primer (CTTGGTCATTTAGAGGAAGTAA) or ITS2 reverse primer (GCTGCGTTCTTCATCGATGC) were used to amplify ITS-1 sequences in triplicate, as previously described [50]. Combined libraries were further purified and sequenced using 454 Titanium chemistry and the 454-GS-FLX sequencing platform (454 Life Sciences, Branford, CT). Since mucosal samples did not consistently yield ITS-1 PCR products, probably due to low fungal load, only saliva samples were evaluated via ITS-1 sequencing.

### Processing of sequences and taxonomic classification

16S rRNA gene reads were processed in mothur [52]. Primers and barcodes were trimmed followed by removal of sequences shorter than 200 bp, with homopolymers greater than eight nucleotides or ambiguous base calls. Sequences were then filtered using a 50 bp sliding window approach and an average quality score threshold of 35 [53]. Chimeric sequences were removed with UChime [54], in mothur. Sequences were then classified to species level by using the classify.seqs command and the Human Oral Microbiome Database (HOMD) V14.5 as reference. Parameters used were: method = knn, search = blast, gapopen = − 5, gapextend = − 5, match = 4, mismatch = − 5, numwanted = 1, following recommendations by Al-Hebshi et al. [55]. This taxonomy assignment algorithm was validated by classifying HOMD reference sequences trimmed to include only the V1–V2 region against the HOMD full-length reference sequence database. With a few exceptions, all V1–V2 short sequences were correctly classified in the validation test. However, the following species could not be discriminated from each other: *Lactobacillus casei* and *Lactobacillus rhamnosus*; *Veillonella parvula* and *Veillonella dispar*; *Streptococcus mitis*, *Streptococcus pneumoniae*, and *Streptococcus* sp. HOT423; and *Neisseria flavescens* and *Neisseria subflava*. Counts for species that could not be correctly identified were aggregated.

ITS-1 reads were also processed in mothur [52]. Sequences were trimmed and quality filtered and chimeras removed as described for 16S rRNA sequences. Sequences were then classified to genus level by using the classify.seqs command and a modified version of the Findley et al. database [56] as a reference, employing the recommended parameters (https://www.mothur.org/wiki/Findley_ITS_Database). Prior to this, a curation of the Findley et al. database was conducted to join synonym taxa under one preferred name [57]. To accomplish this, reference sequences from synonym taxa were compared via BLAST against NCBI’s nucleotide type strain database and against the Fungal Metagenomics Project database. After confirming their genus identity, *Gueomyces* sequences were included under *Trichosporon*; *Lewia* under *Alternaria*; *Valsa* under *Cytospora*; *Coprinellus* and *Coprinopsis* under *Coprinus*; *Erythrobasidium* under *Rhodotorula*; *Cochliobolus* under *Curvularia*; *Filobasidium*, *Cystofilobasidium* and *Dioszegia* under *Cryptococcus*; and *Emericella* under *Aspergillus*. In addition, sequences of *Cladosporium*, *Toxicocladosporium*, *Aureobasidium*, *Kabatiella*, *Scleroconidioma*, and *Candida* were added as they were underrepresented in the database. *Malassezia* and *Candida* ITS-1 sequences were further classified to species level, using curated and aligned reference libraries and the software package pplacer [58], as previously described [56]. A total of 691 saliva- and mucosa-derived libraries were sequenced. All sequences are available at the Short Reads Archive (Accession number PRJNA399163).

### Alpha and beta-diversity estimates for 16S rRNA gene and ITS-1 amplicon data

16S rRNA gene libraries were subsampled at 3074 reads and ITS-1 reads at 1338 reads. Rarefaction curves were generated via the R package vegan and can be found in Additional file 1: Figure S5. Most curves were starting to become asymptotic at the thresholds used for subsampling. Community diversity was evaluated via the non-parametric Shannon Index and community structure via the Yue-Clayton Theta Index (Theta_YC_) as calculated in mothur. These metrics were constructed based on species-level taxonomic units for 16S rRNA gene reads and genus-level for ITS-1 (with the exception of *Malassezia* and *Candida* that were speciated). Mucosal communities were analyzed in two different manners, including a separate analysis of samples according to the clinical status of the sampled site (healthy, erythematous, or ulcerated) and an analysis in which data from different sites within a subject were combined and analyzed as one mucosal unit.

### Evaluation of the antimicrobial activity of chemotherapeutics

To evaluate the effect of antineoplastics on the whole cultivable salivary microbiota, unstimulated saliva from eight subjects was collected in sterile tubes. Aliquots were incubated with 5-FU (7.7 μM), 5-FU (7.7 μM) + docetaxel (1.2 μM), or a vehicle control, at 37 °C for 2 h. Samples were serially diluted and plated on solid media containing tryptic soy agar (20 g/L), brain heart infusion agar (26 g/L), yeast extract (10 g/L), sheep blood (5% vol/vol), hemin (5 μg/mL), menadione (0.3 μg/mL), and *N*-acetyl muramic acid (10 ug/mL) for enumeration of salivary bacteria [59]. Sets of duplicate plates were incubated either anaerobically or aerobically at 37 °C for 5 days.

To evaluate the effect of 5-FU on pure bacterial cultures, microorganisms were grown in appropriate medium and atmosphere under late logarithmic phase and this culture was diluted in fresh medium to an OD_(600 nm)_ = 0.4. Normalized culture aliquots were incubated with different concentrations of 5-FU or with a vehicle control for 2 h, at 37 °C, under an appropriate atmosphere, after which cultures were serially diluted and plated for colony forming unit determination. The effect of 5-FU on the growth of *S. salivarius* ATCC 9222 and *F. nucleatum* ATCC 49256 was determined by adding 5-FU or vehicle control to starter cultures diluted to an OD = 0.1. Growth was followed until early stationary phase. Inhibition was determined by comparing the area under the curve of test conditions compared to control vehicle. All experiments were repeated three times. Concentrations tested of 5-FU and docetaxel in the above experiments were based on their bioavailability in plasma and oral fluids [59, 60].

### Changes in global oral epithelial gene expression during chemotherapy

RNA was isolated from cytologic smears using the miRNeasy Mini Kit (Qiagen). RNA quantity and quality were measured using the Experion High Sensitivity RNA Kit. As previously observed by others [61], RNA quantity and quality from oral epithelium varies with some samples showing very low yields and high degradation. Therefore, we employed the whole genome cDNA-mediated Annealing Selection Extension and Ligation assay (wgDASL) in combination with the HumanHT-12 v4 Expression Bead Chip (Illumina), to evaluate gene expression. This assay performs well with partially degraded RNA as it does not depend on an intact poly-A tail for cDNA synthesis [62]. Only subjects with matched baseline and V3 samples that yielded at least 500 ng of RNA were included in the analysis. The wgDASL assay was performed with 250 ng of RNA, including two technical replicates. Initial data analysis to eliminate samples that did not perform well in the assay was carried out using GenomeStudio (Illumina). Data were first normalized using the quantile method, followed by filtering out probes without a detection *p* value < 0.05 in at least one sample. Outlier samples for which technical replicates did not have a correlation of at least *r* = 0.95 were removed from the dataset. Differential gene expression analysis in 14 subject-paired samples from baseline to V3 was then performed using Significance Analysis of Microarrays (SAM) [63]. Gene ontology enrichment analysis was performed using DAVID [64], and redundant GO terms summarized and visualized using REVIGO [65].

### Effect of commensals on a 3D oral mucosa analog tissue

To evaluate the response of the oral mucosa to microbial commensals, 3D multi-layered epithelial cell constructs derived from primary keratinocytes (EpiOral™, MatTek Corporation) were challenged with 10^8^ bacterial cells per cm^2^. Microorganisms were placed apically and incubated with tissues for 24 h. Membranes containing tissues were harvested and used for histologic sectioning or RNA extraction. Cell death was evaluated in tissue sections via the DeadEnd™ Fluorometric TUNEL System (Promega). RNA was extracted via Qiagen’s RNeasy mini kit, reverse transcribed using the High Capacity RNA-to-cDNA Kit (Applied Biosystems), and evaluated via real-time PCR using TaqMan Assays (Applied Biosystems). The 18S rRNA gene was used as endogenous control.

### Statistical analyses

Baseline data were compared between groups by either chi-square or Fisher’s exact test, the latter applied when more than 20% of table cell counts had expected frequencies < 5. Continuous data were evaluated via independent sample *t* tests or Mann-Whitney *U* tests, according to data distribution. The distribution of continuous data was tested for normality using measures of Skewness and Kurtosis and the Shapiro-Wilk test for normality in SPSS. Differences in mucositis incidence across regimens were evaluated via chi-square. Longitudinal data were evaluated in subject-matched samples. Of interest was how changes in one variable were associated with changes in another variable or with one time-point measures. Specifically, we were interested in the longitudinal analysis of changes in mucositis scores and the relationship of these changes with other variables. We therefore modelled the change in each longitudinal variable using linear or quadratic orthogonal polynomial contrasts. For this, each data point was multiplied by a standard coefficient (for a linear or quadratic 4 level contrast) and data aggregated by subject. Correlations between linear or quadratic longitudinal change and other variables were determined via Spearman Rank tests. Differences in clinical variables and microbiome diversity from baseline to each visit were calculated using paired-Wilcoxon Rank tests. The change in alpha-diversity (measured with the non-parametric Shannon Index) was also evaluated by subtracting values at each visit from the respective baseline values. Correlations between the difference in non-parametric Shannon Index values and other variables were evaluated via Spearman Rank tests. To quantify changes in community structure during chemotherapy, we calculated the Theta_YC_ distance of each sample to its corresponding baseline and compared distances to control subjects via Mann-Whitney Rank tests. Theta_YC_ distances were also evaluated for their correlation with demographic and clinical variables via Spearman Rank tests. Linear regression analysis was used to evaluate the contribution of different predictors to alpha- and beta-diversity microbiome change. Changes in salivary community structure during chemotherapy and correlation of these changes with clinical variables and microbiome diversity were also visualized in Principal Coordinates Analysis (PCoA) plots based on the Theta_YC_ distances. Significant separation between data clouds was tested via AMOVA and the correlations of principal components with metadata were evaluated via Spearman Rank tests. Differences in relative abundances of individual microbial taxa from baseline to each visit were evaluated via Wilcoxon signed rank tests. The significance threshold for all statistical tests mentioned above was adjusted using the Benjamini Hochberg false discovery rate method. Evaluation of the longitudinal covariation in microbial relative abundances and clinical parameters measured at the four visits of the study was performed using the R package MixOmics [66]. For this analysis, we used the multilevel function and compared microbial and clinical variables using sparse Partial Least Squares (sPLS). This multilevel multivariate approach takes into account the repeated measures within subjects and by using sPLS reveals covariation patterns in all variables in an unsupervised manner. Differences in colony forming units (CFUs) recovered after treatment of saliva or pure bacterial cultures with antineoplastics were determined via paired Wilcoxon signed rank tests. Differences in growth of bacteria in the presence and absence of 5-FU were determined by comparing the area under the growth curve for different conditions via *t* tests.

## Additional files

Additional file 1: **Figure S1.** Study design. **Figure S2.** Effect of chemotherapy on the oral microbiome community structure. **Figure S3.** Taxa that changed in relative abundance at each study visit compared to baseline levels. **Figure S4.** Effect of antineoplastics on the oral microbiome. **Figure S5.** Rarefaction curves for all bacterial and fungal amplicon libraries. (PDF 9205 kb)

Additional file 2: **Table S1.** Demographic and baseline clinical characteristics of study participants. **Table S2.** Descriptive statistics specific to the cancer cohort. **Table S3.** Correlations between change in oral mucositis severity (OMAS) or change in salivary bacterial diversity with demographic and clinical characteristics. **Table S5.** Sequencing summary. **Table S6.** Within subject differences in alpha-diversity (non-parametric Shannon index) between mucosal sites with different clinical appearance. **Table S7.** Main clinical characteristics of subjects included in the mucosal gene expression analyses. (PDF 221 kb)

Additional file 3: **Table S4.** Identity and proportions of taxa with significantly different relative abundance at any study visit compared to baseline. (XLSX 15 kb)

Additional file 4: **Table S8.** SAM analysis of differential gene expression between baseline and V3 in 14 subjects, showing 158 upregulated and 2 downregulated genes at V3 (> 1.5-fold). (XLSX 24 kb)

Additional file 5: **Table S9.** Summary of upregulated gene ontology (GO) term categories. (XLSX 13 kb)

## Abbreviations

5-FU: 5-Fluorouracil
CPITN: Community Periodontal Index of Treatment Needs
HOMD: Human Oral Microbiome Database
OMAS: Oral Mucositis Assessment Scale
SFR: Salivary flow rate
sPLS: Sparse Partial Least Squares analysis
Theta_YC_: Yue-Clayton Theta Index
WHO: World Health Organization
