# Supplementary material for: Chemotherapy-induced oral mucositis is associated with detrimental bacterial dysbiosis
Source: Microbiome. 2019 Apr 25;7:66. doi: 10.1186/s40168-019-0679-5 (PMC6482518; doi:10.1186/s40168-019-0679-5)
Supplement: Supplementary file 1 — Figure S1. Study design. Figure S2. Effect of chemotherapy on the oral microbiome community structure. Figure S3. Taxa that changed in relative abundance at each study visit compared to baseline levels. Figure S4. Effect of antineoplastics on the oral microbiome. Figure S5. Rarefaction curves for all bacterial and fungal amplicon libraries. (PDF 9205 kb) [file 40168_2019_679_MOESM1_ESM.pdf]

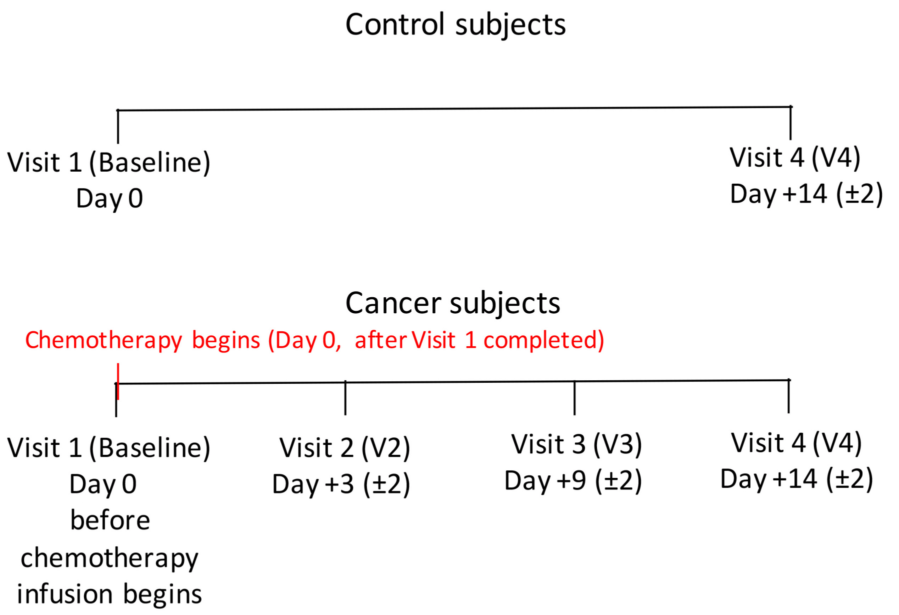

Controls	Cancer
**Inclusion criteria**<br>• Able and willing to provide written informed consent<br>• 21 years of age or older<br>**Exclusion criteria**<br>• Primary diagnosis of systemic disease within one month preceding study participation<br>• Use of systemic antibiotics within 2 months before baseline visit<br>• Initiation of use of any new medication (over the counter or prescription) taken on regular basis (“regularly” defined as > 3 doses per week) within one month preceding, or during, study participation<br>• Diagnosed with HIV<br>• Use of inhaled corticosteroids<br>• History of cancer treated with systemic chemotherapy or radiation to the head and neck<br>• Presence of oral mucosal disease including lichen planus, desquamative gingival conditions or open oral ulceration<br>• Clinically documented oral fungal or viral infection of the oral cavity<br>• Planned dental procedures during period of study participation<br>• Women who are pregnant<br>• Use of commercial probiotic supplements	**Inclusion criteria**<br>• Able and willing to provide written informed consent<br>• 21 years of age or older<br>• Diagnosed with a solid tumor and planning to receive 5-FU- or Adriamycin-based chemotherapy<br>**Exclusion criteria**<br>• Tumors of the craniofacial region directly involving the oral cavity<br>• Use of antibiotics, antifungals or antimicrobial mouthrinses within 48 hours before baseline visit<br>• Presence of oral mucosal disease including oral mucositis, lichen planus, or desquamative gingival conditions<br>• Clinically documented fungal or viral infection of the oral cavity

Figure S1. Study design. Top diagram shows visit intervals in which controls and cancer subjects were evaluated. Day 0 in the cancer group was the day of the first infusion, with baseline data collected prior to subjects being infused. Number in parentheses indicates range of days within which each visit occurred. Bottom Table shows inclusion and exclusion criteria for the two cohorts.

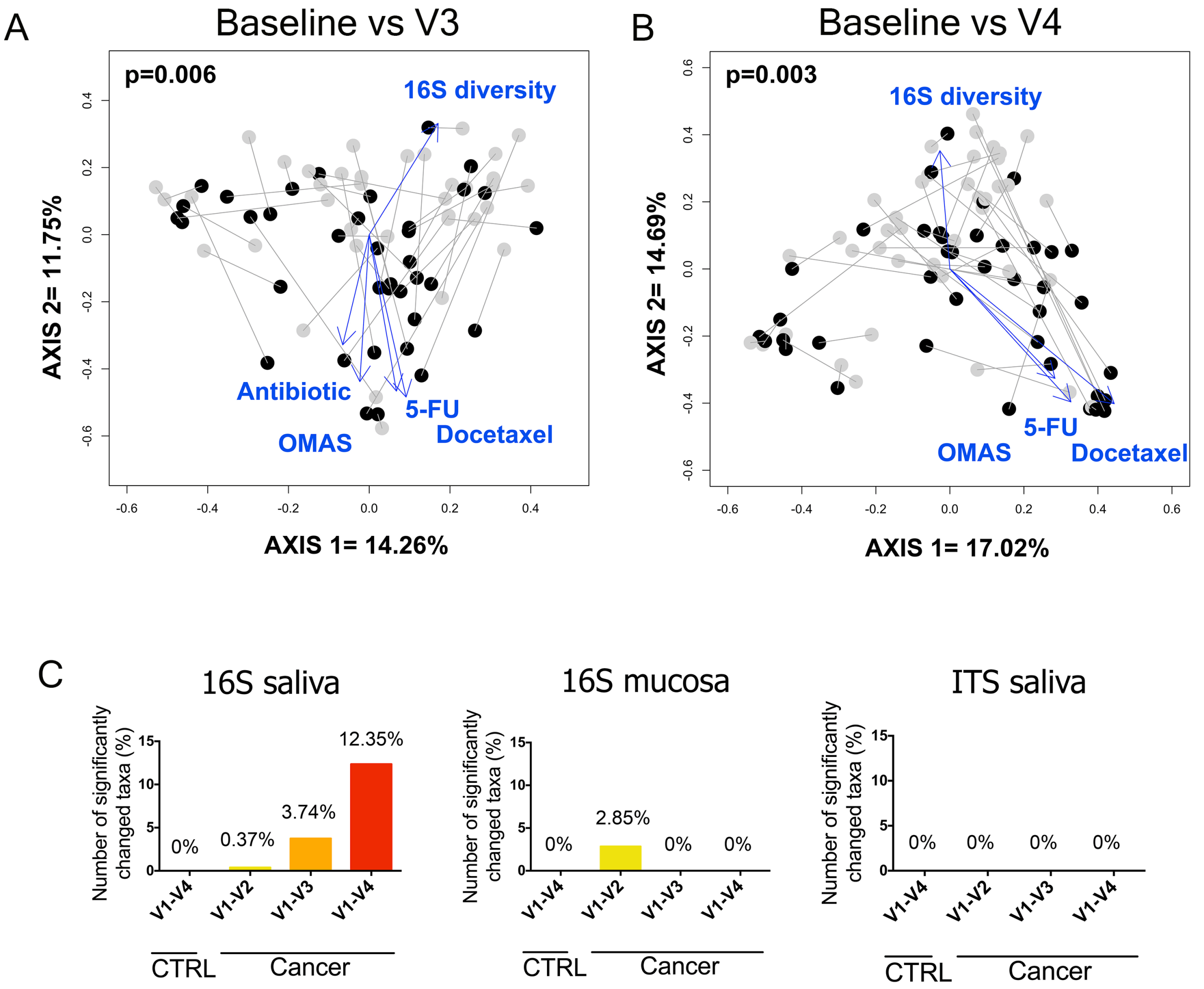

Figure S2. Effect of chemotherapy on the oral microbiome community structure. Panels A and B show Principal Coordinates Analysis (PCoA) plots based on the Theta$_{YC}$ distances showing changes in salivary bacterial community structure during chemotherapy and correlation of principal components with changes in bacterial alpha-diversity, mucositis severity, chemotherapeutic drug doses and antibiotic intake. Baseline samples appear in light gray, and samples collected during chemotherapy (V3 or V4) in black. Plot A shows changes from baseline to V3 and plot B shows changes from baseline to V4. Lines join subject-paired samples. Significant separation between data clouds (baseline to V3 or baseline to V4) was tested via AMOVA and P

values for this test are depicted in the upper left corner of each plot. Blue vector arrows indicate metadata correlated with axes (Spearman), and therefore associated with microbiome variability. Vector arrows were plotted from the origin using correlation coefficients with axes as coordinates and therefore longer vector arrows indicate stronger correlations. 16S diversity = change in the non-parametric Shannon diversity Index from baseline to V3 or V4; 5-FU = total dose received of 5-FU; docetaxel = total dose received of docetaxel. Antibiotic = intake of a multi-dose antibiotic prior to the visit; OMAS = mucositis severity score at each visit. Panel C shows percentage of taxa from each community that significantly changed in relative abundance from baseline to each visit. Salivary bacterial communities show the greatest number of significantly changed taxa as chemotherapy progressed. For these determinations, taxa relative abundances were compared via paired Wilcoxon Signed Rank tests and significance threshold adjusted via the FDR method.

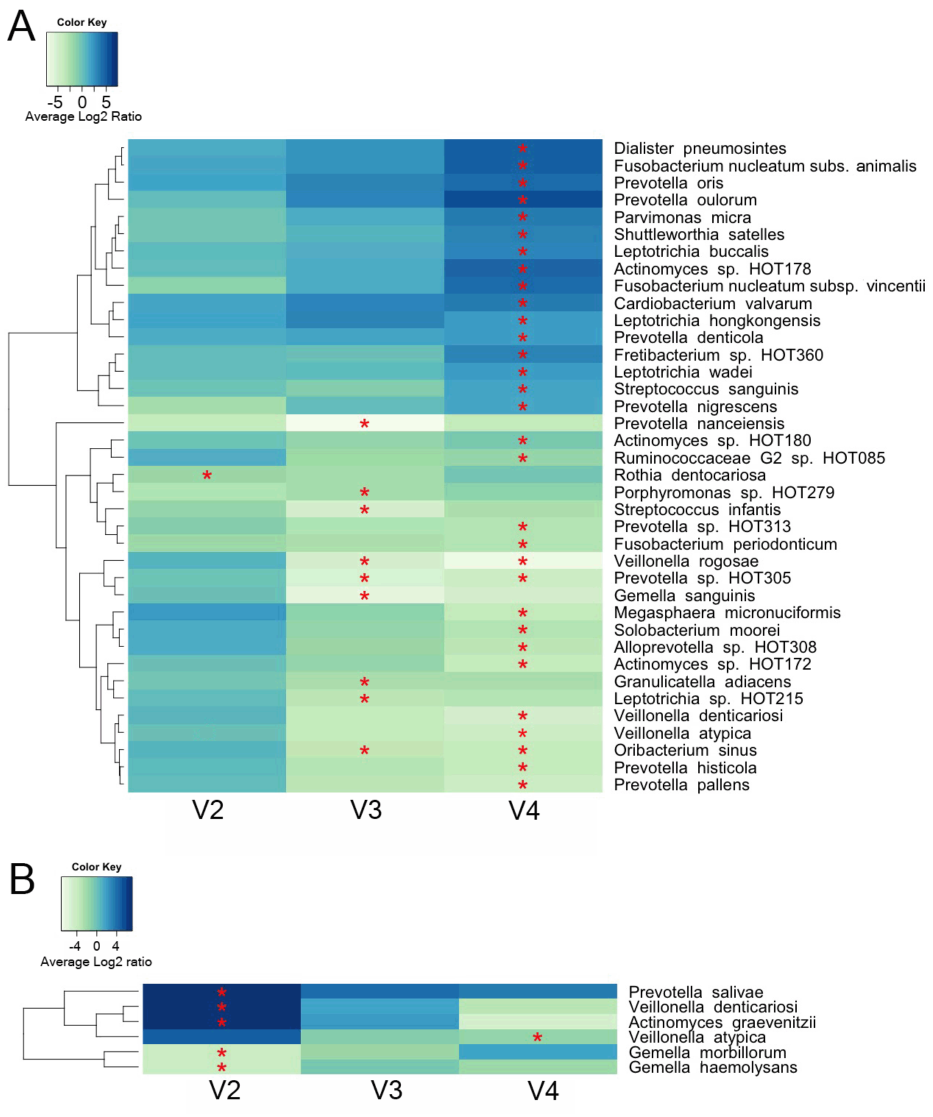

Figure S3. Taxa that changed in relative abundance at each study visit compared to baseline levels. Heatmaps depict the average log2 fold-change with red asterisks indicating taxa that changed significantly as evaluated via paired sample Wilcoxon Rank tests adjusted for multiple testing. Panel A depicts taxa that changed in saliva and panel B depicts taxa that changed in mucosa.

A

		5-FU	Docetaxel	Cisplatin	Doxorubicin
Saliva	Streptococcus salivarius	-0.486(0.004)	-0.480(0.004)	-0.338(0.051)	0.475(0.005)
	Streptococcus parasanguinis II	-0.483(0.004)	-0.450(0.008)	-0.230(0.191)	0.361(0.036)
	Granulicatella adiacens	-0.467(0.005)	-0.334(0.053)	-0.227(0.197)	0.298(0.087)
	Actinomyces graevenitzii	-0.519(0.008)	-0.446(0.025)	-0.431(0.032)	0.486(0.014)
	Veillonella rogosae	-0.409(0.023)	-0.583(0.001)	-0.268(0.145)	0.416(0.020)
	Veillonella atypica	-0.350(0.046)	-0.558(0.001)	-0.191(0.287)	0.350(0.046)
	Veillonella denticariosi	-0.454(0.013)	-0.508(0.005)	-0.269(0.158)	0.364(0.052)
	Prevotella pallens	-0.299(0.156)	-0.585(0.003)	-0.581(0.003)	0.229(0.282)
	Leptotrichia sp. HOT 215	-0.394(0.046)	-0.557(0.003)	-0.317(0.115)	0.114(0.579)
	Fusobacterium nucleatum subsp. vincentii	0.272(0.154)	0.375(0.045)	0.288(0.129)	-0.430(0.020)
Mucosa	Streptococcus parasanguinis II	-0.228(0.262)	-0.552(0.001)	-0.351(0.079)	0.146(0.478)
	Actinomyces graevenitzii	-0.512(0.003)	-0.411(0.090)	-0.366(0.135)	0.482(0.043)
	Veillonella rogosae	-0.552(0.010)	-0.760(<0.001)	-0.529(0.014)	0.508(0.019)
	Prevotella oris	0.594(0.004)	0.266(0.244)	0.420(0.058)	-0.417(0.060)

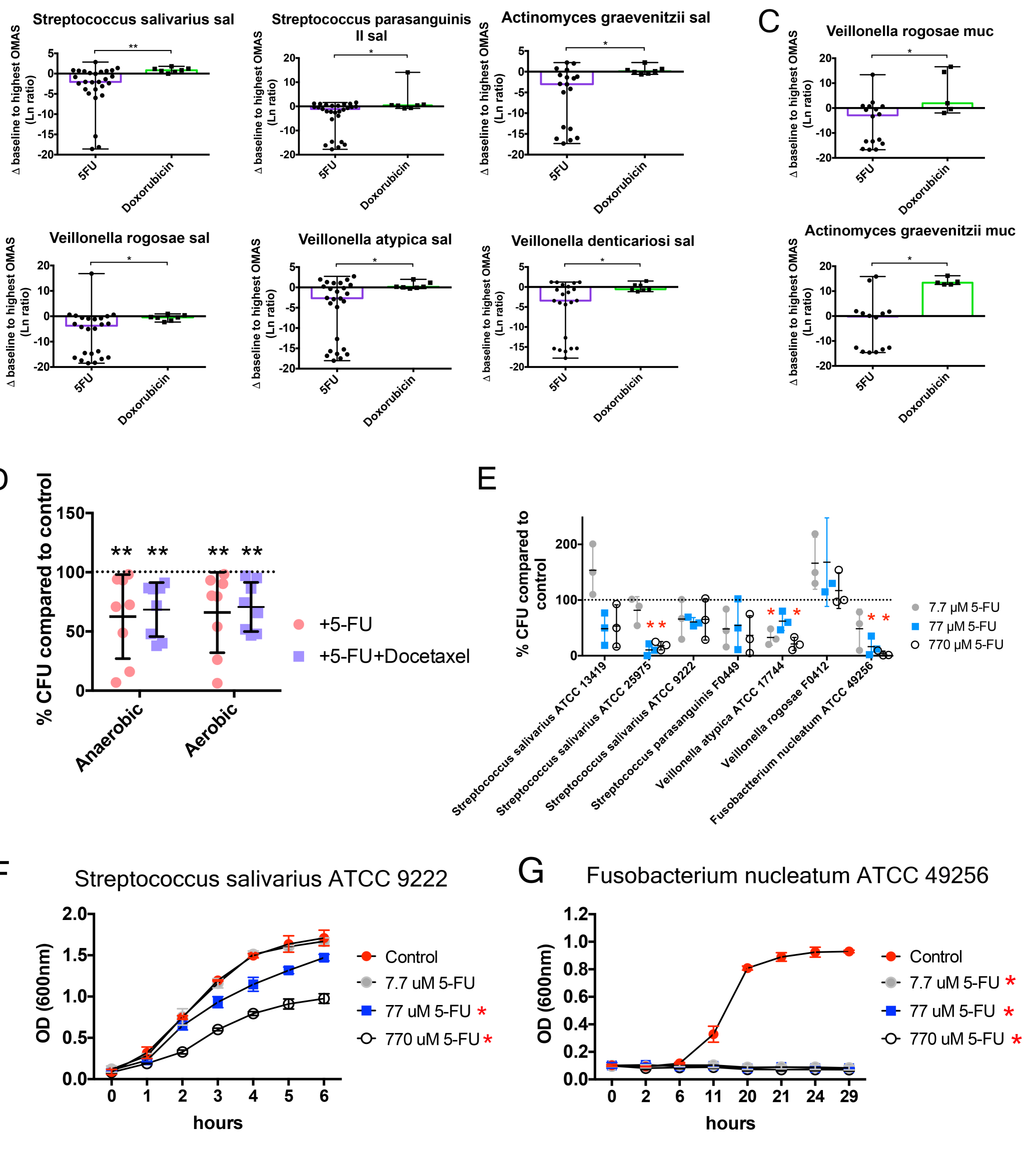

Figure S4. Effect of antineoplastics on the oral microbiome. Panel A shows correlations between changes in relative abundance of salivary and mucosal bacterial species and antineoplastic doses. Data evaluated for a correlation were the log_2 change in relative abundance of each species from baseline to the visit with the most severe mucositis and the total dose of antineoplastics. All species identified as correlated with mucositis in Fig. 4 were evaluated but only those also showing significant correlations with antineoplastic doses are shown. Data represent Spearman correlation coefficients with p values in parenthesis. Colored cells show correlations significant after adjustment for multiple comparisons via the FDR method. Panels B and C show species that followed a different direction of change in subjects taking 5-FU and subjects receiving doxorubicin in saliva (B) or mucosa (C). Graphs show change in proportions from baseline to the visit with the most severe mucositis. * indicates a p value <0.05 and ** a p value <0.01 when comparing the change between regimen groups via Mann-Whitney Rank tests. Panel D shows the effect of exposure of salivary communities (n=8 subjects) to 7.7 µM 5-FU alone or in combination with 1.2 µM docetaxel. Data represent percentage of total colony forming units (CFUs) detected after a 2 hour exposure compared to a vehicle control. * indicates a p value <0.05 when control and test groups were compared via paired Wilcoxon rank tests. Panel E shows the effect of different 5-FU concentrations on viability of bacterial commensals in pure culture after 2 hours exposure. Data represent percentage of total colony forming units (CFUs) detected after a 2 hour exposure compared to a vehicle control. * indicates a p value <0.05 when control and test groups were compared via paired Wilcoxon rank tests. Panels F and G show the effect of different concentrations of 5-FU on the growth of *Streptococcus salivarius* or *Fusobacterium nucleatum*. * indicates area under the curve is significantly different between control and test conditions (p<0.05).

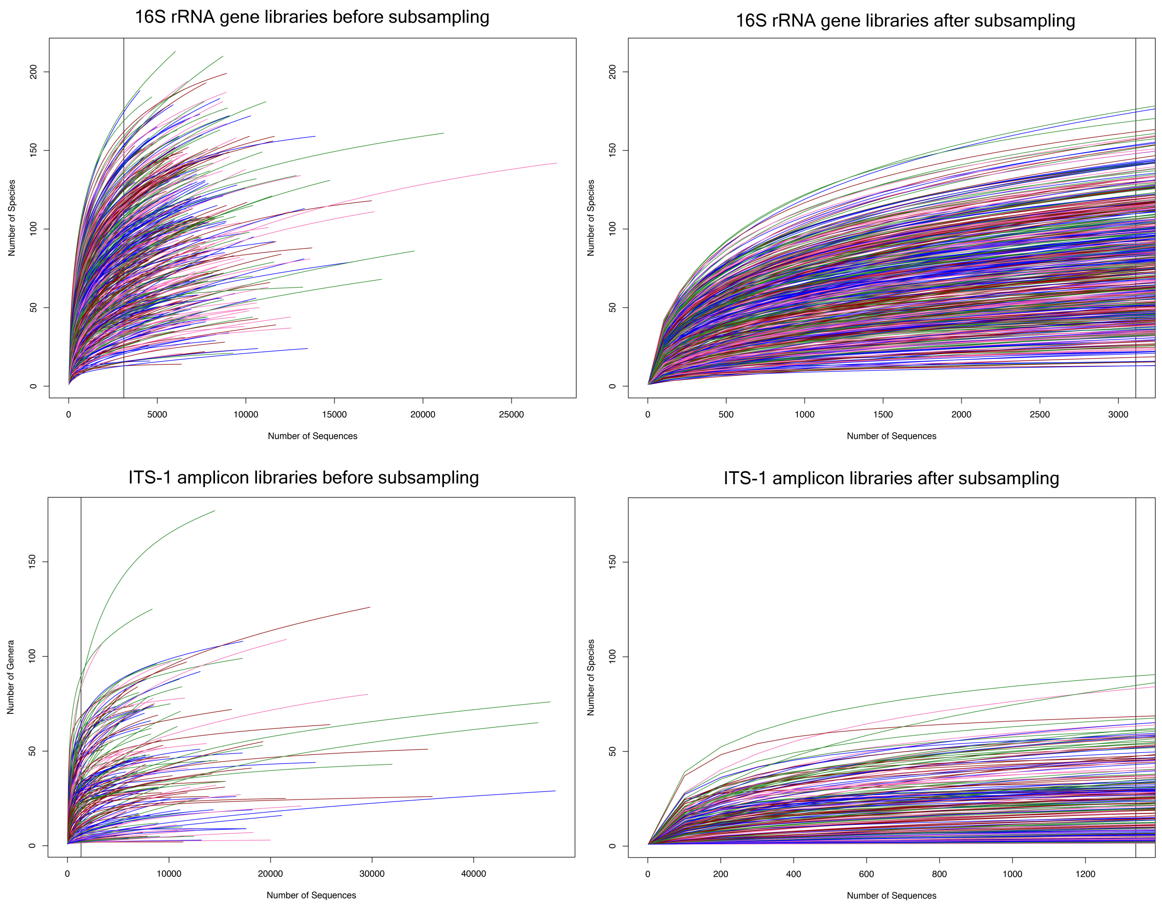

Figure S5. Rarefaction curves for all bacterial and fungal amplicon libraries. Plot in the left depicts sequencing depth and plot in the right shows rarefaction curves after imposed subsampling thresholds (vertical black lines).
