# Supplementary material for: Chemotherapy-induced oral mucositis is associated with detrimental bacterial dysbiosis
Source: Microbiome. 2019 Apr 25;7:66. doi: 10.1186/s40168-019-0679-5 (PMC6482518; doi:10.1186/s40168-019-0679-5)
Supplement: Supplementary file 2 — Table S1. Demographic and baseline clinical characteristics of study participants. Table S2. Descriptive statistics specific to the cancer cohort. Table S3. Correlations between change in oral mucositis severity (OMAS) or change in salivary bacterial diversity with demographic and clinical characteristics. Table S5. Sequencing summary. Table S6. Within subject differences in alpha-diversity (non-parametric Shannon index) between mucosal sites with different clinical appearance. Table S7. Main clinical characteristics of subjects included in the mucosal gene expression analyses. (PDF 221 kb) [file 40168_2019_679_MOESM2_ESM.pdf]

Table S1. Demographic and baseline clinical characteristics of study participants.

Variable	Non-cancer n=30	Cancer n=49			Statistic (Non-cancer vs All cancer)
		Chemotherapy -naïve n=32	Chemotherapy -exposed n=17	All Cancer n=49	
**Age**	**47.5±14.1**	58.7±13.9	56.6±10.3	**58.1±13.1**	**0.002***
**Gender (% male)**	**30.0**	**68.8$^{\Psi}$**	**29.4$^{\Psi}$**	**55.1**	**0.030#**
Race (% white)	100.0	90.6	88.2	89.8	0.132@
Ethnicity (% hispanic)	6.7	6.3	5.9	6.1	1.000@
Smoking history (% yes)	47.8&	68.8	52.9	63.2	0.226#
Former smoker (% yes)	39.1&	53.1	41.2	48.8	0.457#
Current smoker (% yes)	8.7&	15.6	11.8	14.3	0.700@
Acid inhibitor use (% yes)	6.7	18.8	41.2	26.5	0.103@
Inhaler steroid use (% yes)	0.0	6.3	5.9	6.1	0.288@
**Number of teeth**	**28(0-32)**	25(0-32)	28(0-32)	**26(0-32)**	**0.001^**
Prosthetic teeth (% yes)	66.7	78.1	82.4	79.6	0.285#
Number of teeth replaced by prosthesis	1(0-28)	2(0-32)	2(0-32)	2(0-32)	0.233^
Removable prosthesis (% yes)	6.7	15.6	11.8	14.3	0.453@
Visible caries lesions (% yes)	23.3	37.5	17.6	30.6	0.438@
Number of teeth with visible caries lesions	0(0-2)	0(0-11)	0(0-4)	0(0-11)	0.267^
**Plaque index**	**0.6(0.1-1.3)**	1.1(0.0-2.5)	1.1(0.6-1.8)	**1.1(0.0-2.5)**	**0.001^**
Periodontal status (CPITN, %)					0.303@
• Healthy	16.7	6.3	0.0	4.1	
• Bleeding	0.0	3.1	0.0	2.0	
• Calculus	30.0	31.3	47.1	36.7	
• Periodontal pocket between 4 and 5.5 mm	46.7	37.5	41.2	38.8	
• Periodontal pocket > 5.5 mm	3.3	15.6	5.9	12.2	
• Edentulous	3.3	6.3	5.9	6.1	
Salivary flow rate (mL/min)	0.4(0.1-1.2)	0.4(0.1-1.5)	0.3(0.1-0.9)	0.4(0.1-1.5)	0.705^
**Subjective oral dryness (1 to 10 scale)**	**2(0-6)**	5(0-8)	4(0-9)	**4(0-9)**	**0.009^**
**Peripheral absolute neutrophil count (x 1000/mm^3 blood)**	**3.1(1.5-5.4)**	5.9(2.2-18.4)	7.3(2.5-13.9)	**6.1(2.2-18.4)**	**<0.001^^{f}**
Oral neutrophil count (x 10,000/rinse)	62(5-203)	46(6-229)	38(6-473)	43(6-473)	0.283^

The distribution of continuous data was tested for normality using measures of Skewness and Kurtosis and the Shapiro-Wilk test in SPSS. For normally-distributed continuous variables mean ± standard deviation is shown. For non-normally distributed continuous variables median is shown with range in parenthesis. Statistical tests for continuous data were applied according to data distribution. For non-continuous variables data shown represent percentage of subjects positive. &Smoking data in the non-cancer group are based on 23 subjects as 7 subjects did not provide this information. *Independent sample t-test; #chi-square; @Fisher's exact test; ^Mann-Whitney U test. $^{\Psi}$Chemotherapy-naïve and chemotherapy-exposed subjects did not significantly differ in any parameter, except for gender. ^{f}Blood neutrophil counts were higher at baseline in cancer subjects since some individuals received steroids, known to induce neutrophilia, days prior to commencing chemotherapy.

Table S2. Descriptive statistics specific to the cancer cohort.

	Variable	All subjects with cancer	Chemotherapy-naïve	Chemotherapy-exposed
Diagnosis	Squamous-cell carcinoma	42.9%	56.3%	17.6%
	Breast cancer	28.6%	18.8%	47.1%
	Adenocarcinoma	22.4%	18.8%	29.4%
	Other cancer	6.1%	6.3%	5.9%
Chemotherapeutic drugs received during cycle	5-FU	69.4%	81.3%	47.1%
	Doxorubicin	30.6%	18.8%	52.9%
	Docetaxel	42.9%	56.3%	17.6%
	Cyclophosphamide	28.6%	18.8%	47.1%
	Cisplatin	34.7%	43.8%	17.6%
	Carboplatin	14.3%	18.8%	5.9%
	Oxaliplatin	14.3%	12.5%	17.6%
	Leucovorin	16.3%	12.5%	23.5%
	Herceptin	4.1%	3.1%	5.9%
	Mitomycin	4.1%	3.1%	5.9%
	Bevacizumab	4.1%	0%	11.8%
	Irinotecan	2%	0%	5.9%
	Vinblastine	2%	0%	5.9%
Chemotherapy regimen (drug combinations)	5FU-docetaxel-cisplatin	30.6%	40.6%	11.8%
	5FU-docetaxel-carboplatin	12.2%	15.6%	5.9%
	5FU-oxaliplatin-leucovorin	8.2%	9.4%	5.9%
	5FU	4.1%	6.3%	0.0%
	5FU-mitomycin	4.1%	3.1%	5.9%
	5FU-oxaliplatin-leucovorin-herceptin	4.1%	3.1%	5.9%
	5FU-cisplatin-carboplatin	2.0%	3.1%	0.0%
	5-FU-irinotecan-Bevacizumab-leucovorin	2.0%	0.0%	5.9%
	5FU-oxaliplatin-leucovorin-Bevacizumab	2.0%	0.0%	5.9%
	Doxorubicin-cyclophosphamide	28.6%	18.8%	47.1%
	Doxorubicin-cisplatin-vinblastine	2.0%	0.0%	5.9%
Antibiotic use	Any antibiotic	40.8%	53.1%	17.6%
	Single dose prophylactic	22.4%	34.4%	0%
	Multi-dose	20.4%	21.9%	17.6%
Steroid	Steroid pre-chemotherapy	18.4%	18.8%	17.6%
	Steroid during cycle	75.5%	81.3%	64.7%
Other	Pegfilgrastim	73.5%	75.0%	70.6%
	Acid inhibitors	69.4%	81.3%	47.1%

Table S3. Correlations between change in oral mucositis severity (OMAS) or change in salivary bacterial diversity with demographic and clinical characteristics.

	Change vs zero (p value)	Oral mucositis (OMAS) (Q)	Oral mucositis (OMAS) (L)	Salivary bacterial diversity (Q)	Salivary bacterial diversity (L)
Oral mucositis (Q)	<0.001	-	-0.350(0.016)	-0.070(0.659)	0.076(0.621)
Oral mucositis (L)	<0.001	-0.350(0.016)	-	0.013(0.935)	-0.192(0.201)
Salivary bacterial diversity (Q)	0.051	-0.070(0.659)	0.013(0.935)	-	-0.071(0.653)
Salivary bacterial diversity (L)	0.009	0.076(0.621)	-0.192(0.201)	0.071(0.653)	-
Salivary flow rate (Q)	0.742	0.028(0.850)	0.040(0.789)	0.066(0.680)	0.115(0.452)
Salivary flow rate (L)	0.002	-0.481(0.001)	0.419(0.003)	-0.045(0.776)	-0.005(0.974)
Plaque Index (Q)	0.767	0.230(0.129)	-0.180(0.237)	0.047(0.772)	0.053(0.734)
Plaque Index (L)	0.940	-0.226(0.135)	0.318(0.033)	0.125(0.442)	-0.037(0.815)
Oral neutrophil count (Q)	0.398	0.023(0.878)	-0.057(0.702)	-0.152(0.388)	0.249(0.099)
Oral neutrophil count (L)	0.623	-0.140(0.350)	0.385(0.007)	-0.084(0.597)	0.123(0.422)
Peripheral neutrophil count (Q)	<0.001	-0.207(0.168)	0.463(0.001)	0.254(0.108)	-0.064(0.679)
Peripheral neutrophil count (L)	0.360	0.034(0.822)	0.332(0.023)	-0.013(0.934)	-0.173(0.254)
Age	-	-0.151(0.312)	0.053(0.716)	0.039(0.805)	0.084(0.581)
Gender (female)	-	0.256(0.082)	-0.267(0.063)	-0.174(0.271)	0.165(0.272)
Ethnicity (white)	-	0.145(0.332)	-0.163(0.264)	-0.116(0.463)	-0.023(0.879)
Number of natural teeth	-	0.245(0.097)	-0.054(0.714)	-0.224(0.153)	-0.124(0.410)
Number of prosthetic teeth	-	-0.427(0.003)	0.038(0.795)	0.005(0.977)	0.224(0.135)
Removable prosthesis	-	-0.437(0.002)	0.118(0.418)	0.120(0.450)	0.215(0.151)
Number of teeth with caries lesions	-	0.032(0.830)	0.265(0.066)	0.287(0.066)	-0.174(0.248)
Presence of severe periodontitis	-	-0.249(0.099)	-0.064(0.672)	-0.180(0.267)	0.220(0.156)
Smoking history	-	0.090(0.547)	0.349(0.014)	-0.077(0.627)	-0.033(0.830)
Number of years smoked	-	-0.046(0.762)	0.385(0.007)	0.050(0.757)	0.004(0.980)
Current smoker	-	0.149(0.319)	0.349(0.014)	0.217(0.167)	-0.129(0.394)
Antibiotics (any)	-	-0.072(0.632)	0.043(0.771)	-0.180(0.255)	-0.217(0.147)
Multi-dose antibiotic	-	0.017(0.910	0112(0.444)	-0.111(0.482)	-0.446(0.002)
Single-dose antibiotic	-	-0.141(0.343)	-0.134(0.359)	-0.169(0.285)	0.102(0.502)
Steroids	-	0.214(0.149)	0.423(0.002)	0.081(0.611)	-0.100(0.509)
Acid inhibitors	-	-0.182(0.220)	0.247(0.086)	0.340(0.028)	-0.299(0.044)
Doxorubicin-based regimen	-	0.271(0.066)	-0.244(0.091)	-0.143(0.365)	0.206(0.170)
5-FU dose	-	-0.413(0.004)	0.341(0.016)	0.225(0.153)	-0.486(0.006)
Doxorubicin dose	-	-0.226(0.437)	-0.886(<0.001)	0.152(0.619)	0.064(0.822)
Docetaxel dose	-	-0.380(0.008)	0.307(0.032)	0.447(0.003)	-0.266(0.074)
Cyclophosphamide dose	-	0.263(0.074)	-0.342(0.016)	-0.172(0.277)	0.192(0.204)
Cisplatin dose	-	-0.173(0.245)	0.425(0.002)	0.424(0.005)	-0.330(0.025)
Carboplatin dose	-	-0.330(0.023)	0.270(0.061)	-0.108(0.494)	-0.218(0.146)
Oxaliplatin dose	-	0.169(0.257)	-0.325(0.023)	0.068(0.667)	0.082(0.587)
Leucovorin	-	0.186(0.210)	-0.298(0.038)	-0.028(0.861)	0.174(0.247)

As described in Methods, clinical variables measured at more than 1 time point (top 12 rows) were evaluated using linear (L) or quadratic (Q) orthogonal polynomial contrasts. For quadratic change, a positive means a “U” shaped curve (i.e., high to low to high); negative means “inverted U” shape (i.e., low to high to low). For linear change a positive was defined as an upward change while negative was a downward change from baseline. Correlations between longitudinal change and other variables were evaluated via Spearman Rank Correlation tests. Data shown are correlation coefficients with p values in parenthesis. Significance thresholds for each outcome variable (per column) were adjusted for multiple comparisons via the FDR method. Values in red indicate correlations significant after FDR adjustment. Values in yellow indicate correlations with a p value <0.05 but not significant after FDR adjustment.

Table S5. Sequencing summary

		Number of samples	Number of Reads after Pre-processing (median with range)
Cancer	16S saliva	173	6,144 (3,074-13,733)
	16S mucosa healthy	166	6,038 (3,077-27,550)
	16S mucosa erythematous	13	5,972 (3,594-17,098)
	16S mucosa ulcerated	40	6,077 (3,111-10,575)
	Fungal ITS saliva	148	7,993 (1,338-48,056)
	Total Cancer	540	
Control	16S saliva	58	6,516 (4,031-13,919)
	16S mucosa healthy	47	6,781 (3,335-12,453)
	Fungal ITS saliva	46	8,198 (2,045-35,928)
	Total Control	151	
	Total	691	

Table S6. Within subject differences in alpha-diversity (non-parametric Shannon index) between mucosal sites with different clinical appearance.

Groups tested	P value (paired Wilcoxon Rank test)
Healthy mucosa vs erythematous at V2	0.180
Healthy mucosa vs erythematous at V3	0.465
Healthy mucosa vs erythematous at V4	0.053
Healthy mucosa vs ulcerated at V2	0.465
Healthy mucosa vs ulcerated at V3	0.149
Healthy mucosa vs ulcerated at V4	0.799

Table S7. Main clinical characteristics of subjects included in the mucosal gene expression analyses.

Gender	Race	Age	Diagnosis	Regimen	Cycle	Docetaxel	Cyclophosphamide	Cisplatin	Carboplatin	Oxaliplatin	Leucovorin	Cytoxan	Current smoker	OMAS at V3
Male	White	77	Squamous cell carcinoma	5-FU-based	1st	yes	no	no	yes	no	no	no	no	2
Male	White	56	Squamous cell carcinoma	5-FU-based	2nd	yes	no	yes	no	no	no	no	yes	11
Male	White	50	Squamous cell carcinoma	5-FU-based	1st	yes	no	yes	no	no	no	no	yes	0
Male	Black	67	Invasive adenocarcinoma	5-FU-based	1st	yes	no	yes	no	no	no	no	no	2
Male	Black	80	Carcinoma of the rectum	5-FU-based	1st	no	no	no	no	no	no	no	no	3
Female	White	35	Invasive ductal breast carcinoma	Doxorubicin-based	1st	no	no	no	no	no	no	yes	no	5
Male	White	63	Squamous cell carcinoma	5-FU-based	1st	yes	no	yes	no	no	no	no	no	12
Female	White	65	Infiltrating ductal breast carcinoma	Doxorubicin-based	1st	no	yes	no	no	no	no	no	no	0
Male	White	62	Squamous cell carcinoma	5-FU-based	1st	yes	no	yes	no	no	no	no	no	10
Male	White	82	Adenocarcinoma	5-FU-based	1st	no	no	no	no	no	no	no	no	2
Male	White	50	Squamous cell carcinoma	5-FU-based	2nd	yes	no	yes	no	no	no	no	yes	5
Female	White	42	Adenocarcinoma	5-FU-based	3rd	no	no	no	no	yes	yes	no	no	0
Female	White	45	Invasive ductal breast carcinoma	Doxorubicin-based	2nd	no	yes	no	no	no	no	no	yes	4
Female	White	46	Infiltrating ductal breast carcinoma	Doxorubicin-based	2nd	no	yes	no	no	no	no	no	no	5
